# A comparative transcriptomics and eQTL approach identifies *SlWD40* as a tomato fruit ripening regulator

**DOI:** 10.1093/plphys/kiac200

**Published:** 2022-05-04

**Authors:** Feng Zhu, Sagar Sudam Jadhav, Takayuki Tohge, Mohamed A Salem, Je Min Lee, James J Giovannoni, Yunjiang Cheng, Saleh Alseekh, Alisdair R Fernie

**Affiliations:** Max-Planck-Institut für Molekulare Pflanzenphysiologie, Am Mühlenberg 1, Potsdam-Golm 14476, Germany; National R&D Center for Citrus Preservation, Key Laboratory of Horticultural Plant Biology, Ministry of Education, Huazhong Agricultural University, Wuhan 430070, China; Max-Planck-Institut für Molekulare Pflanzenphysiologie, Am Mühlenberg 1, Potsdam-Golm 14476, Germany; Max-Planck-Institut für Molekulare Pflanzenphysiologie, Am Mühlenberg 1, Potsdam-Golm 14476, Germany; Department of Pharmacognosy and Natural Products, Faculty of Pharmacy, Menoufia University, Menoufia 32511, Egypt; Boyce Thompson Institute for Plant Research, Cornell University, Ithaca, New York 14853, USA; Boyce Thompson Institute for Plant Research, Cornell University, Ithaca, New York 14853, USA; US Department of Agriculture–Agricultural Research Service, Robert W. Holley Center for Agriculture and Health, Ithaca, New York 14853, USA; National R&D Center for Citrus Preservation, Key Laboratory of Horticultural Plant Biology, Ministry of Education, Huazhong Agricultural University, Wuhan 430070, China; Hubei Hongshan Laboratory, Wuhan 430070, China; Max-Planck-Institut für Molekulare Pflanzenphysiologie, Am Mühlenberg 1, Potsdam-Golm 14476, Germany; Center of Plant Systems Biology and Biotechnology, Plovdiv 4000, Bulgaria; Max-Planck-Institut für Molekulare Pflanzenphysiologie, Am Mühlenberg 1, Potsdam-Golm 14476, Germany; Center of Plant Systems Biology and Biotechnology, Plovdiv 4000, Bulgaria

## Abstract

Although multiple vital genes with strong effects on the tomato (*Solanum lycopersicum*) ripening process have been identified via the positional cloning of ripening mutants and cloning of ripening-related transcription factors (TFs), recent studies suggest that it is unlikely that we have fully characterized the gene regulatory networks underpinning this process. Here, combining comparative transcriptomics and expression QTLs, we identified 16 candidate genes involved in tomato fruit ripening and validated them through virus-induced gene silencing analysis. To further confirm the accuracy of the approach, one potential ripening regulator, *SlWD40* (*WD-40 repeats*), was chosen for in-depth analysis. Co-expression network analysis indicated that master regulators such as *RIN* (*ripening inhibitor*) and *NOR* (*nonripening*) as well as vital TFs including *FUL1* (*FRUITFUL1*), *SlNAC4* (*NAM, ATAF1,2*, *and CUC2 4*), and *AP2a* (*Activating enhancer binding Protein 2 alpha*) strongly co-expressed with *SlWD40.* Furthermore, *SlWD40* overexpression and RNAi lines exhibited substantially accelerated and delayed ripening phenotypes compared with the wild type, respectively. Moreover, transcriptome analysis of these transgenics revealed that expression patterns of ethylene biosynthesis genes, *phytoene synthase*, *pectate lyase*, and *branched chain amino transferase 2*, in *SlWD40*-RNAi lines were similar to those of *rin* and *nor* fruits, which further demonstrated that *SlWD40* may act as an important ripening regulator in conjunction with *RIN* and *NOR*. These results are discussed in the context of current models of ripening and in terms of the use of comparative genomics and transcriptomics as an effective route for isolating causal genes underlying differences in genotypes.

## Teaser

A reliable approach based on comparative transcriptomics and eQTL is valuable for ripening regulator identification.

## Introduction

Given that seed dispersal is of major ecological and evolutionary importance for all plants and the fact that fleshy fruit plays a vital role in this process, fruit ripening assumes a central importance in the plant life-cycle. It is well documented that hundreds of genes display altered expression during this process (Karlova et al., 2011; Osorio et al., 2011), and that metabolism also undergoes concurrent dramatic shifts to form fruit quality (Carrari and Fernie, 2006). As one of the most important appearance qualities, the accumulation of carotenoids, when combined with naringenin chalcone tainted yellow peel, forms the reddish color of tomato fruits (*Solanum lycopersicum*) (Ballester et al., 2010; Fernandez-Moreno et al., 2016; Zhu et al., 2018). Among the carotenoid biosynthesis pathway, phytoene synthase (PSY) is the key rate-limiting enzyme of the whole pathway; it catalyzes two molecules of GGPP to form the colorless phytoene. Subsequently, under the catalysis of a series of enzymes, phytoene undergoes dehydrogenation and isomerization reactions to form lycopene, which is the dominant carotenoid of tomato fruit (Bird et al., 1991; Bartley and Scolnik, 1993; Cazzonelli and Pogson, 2010; Chen et al., 2019). In addition, the texture of fruits is affected by the modification of cell walls and pectate lyase (PL), which hydrolyzes pectin and is the most substantially cell wall gene contributor to this process identified to date (Yang et al., 2017). Besides appearance and textural qualities, another important quality aspect is taste, which has been attributed to the sugar/organic acid ratio, and volatile and secondary metabolite accumulation. The key genes underlying the levels of these metabolites have been uncovered by a range of quantitative trait loci (Fridman et al., 2004; Tieman et al., 2006; Schauer et al., 2008; Centeno et al., 2011) and genome-wide association studies (Sauvage et al., 2014; Tieman et al., 2017; Ye et al., 2017; Gao et al., 2019).

Moreover, the considerable metabolic changes are coordinated and mediated by transcription factors (TFs) and epigenome dynamics on the metabolic structural genes’ expression (Centeno et al., 2011; Rohrmann et al., 2011; Giovannoni et al., 2017; Lu et al., 2018; Li et al., 2020). Over the last 50 years, several mutants, such as *ripening-inhibitor* (*rin*), *nonripening* (*nor*), *Never ripe* (*Nr*), and *Colorless nonripening* (*Cnr*) mutations, have been identified as severely impacting the tomato ripening process (Tigchelaar, 1973; Lanahan et al., 1994; Vrebalov et al., 2002; Manning et al., 2006). Among these mutants, *rin* is the one of the most famous ripening delaying mutants substantially lacking the ethylene burst and hindering the color change and softening processes, which results from the repression of the ripening inhibitor-macrocalyx (RIN-MC) chimeric protein (Robinson, 1968; Vrebalov et al., 2002; Ito et al., 2017). The integrated analysis of chromatin immunoprecipitation (ChIP)-chip and transcriptome indicated that RIN can directly induce the expression of the key ripening-related structural and regulator genes, *ACS2/4*, *SGR1*, *PSY*, *Cel2*, *EXP1*, *PAL1*, *C4H*, *LoxC*, *AAT1*, *CNR*, *NOR*, *AP2a*, and itself (Fujisawa et al., 2012, 2013; Irfan et al., 2016). Furthermore, the transcriptional behavior of 1,000 TFs has been established during tomato ripening (Rohrmann et al., 2011) and gene regulator networks have been modeled on the basis of these data (Rohrmann et al., 2012). The stronger acting ripening genes mentioned above were, in contrast, identified in mutant screens intent on isolating strong mutants in order to enhance tomato shelf-life (Tigchelaar, 1973; Lanahan et al., 1994; Vrebalov et al., 2002; Manning et al., 2006). While recent years have resulted in the identification of many additional genes with ripening consequences (Li et al., 2021; Shi et al., 2021), it is probable that additional contributors to this complex process remain to be found. As such, further genome-wide analysis is required to mine the regulator affecting this process and provide more comprehensive knowledge on this process.

Time-series, species/accession specific or tissue specific and comparative transcriptomics studies have previously deciphered gene regulatory networks underlying plant developmental pathways allowing the identification of additional functional genes (Breschi et al., 2017; Chang et al., 2019; Batyrshina et al., 2020; Baranwal et al., 2021). For example, Bolger et al. (2014) compared the transcriptomes of M82 and *Solanum*  *pennellii*, and identified 100 key candidate genes related to salt and drought stress. Additionally, transcriptomics studies on genetic mapping populations have defined expression QTLs (eQTLs) as genomic loci controlling variation in steady state levels of transcript between individuals (Sønderby et al., 2007) of what has subsequently become a well-characterized mapping population (Ofner et al., 2016; Szymanski et al., 2020). During tomato domestication, many phenotypes (such as the leaf structure and ripening process) of wild species, such as *S.*  *pennellii* were under strong selection and were substantially different to that of in the cultivar species *S. lycopersicum*. Based on the eQTL analysis of the 76 introgression lines (ILs) from *S. pennellii* in the background of *S. lycopersicum*, Ranjan et al. (2016) identified important genetic regulators of leaf development on chromosomes 4 and 8. The above-mentioned studies demonstrate the power of comparative transcriptomics in combination with ILs; however, limited studies have been carried out using their integrated approach to mine the genes involved in tomato fruit ripening.

As a distant relative of the cultivated tomato *S. lycopersicum*, *S. pennellii* has many substantially different phenotypes with the cultivated tomato and one of these is the mature fruit morphology. The mature fruit of *S. lycopersicum* is red and soft while the mature fruit of *S. pennellii* is green and hard, which renders this pair the ideal parents to cross and illustrate the genetic landscape of fruit ripening. The core set of 76 *S. pennellii* ILs, which represent whole-genome coverage of *S. pennellii* in overlapping segments in the background of M82, have been widely used to identify the key genes of many traits such as yield and metabolic composition (Semel et al., 2006; Alseekh et al., 2013, 2015). In the present study, to identify key candidates regulating tomato fruit ripening, an integrated comparative transcriptomics and eQTL approach was taken utilizing *S. pennellii* ILs (Eshed and Zamir, 1995). We isolated 16 candidates and provided primary validation of eight of them as being involved in the ripening process via the virus-induced gene silencing (VIGS) method. Following this screen, one candidate, *SlWD40*, was taken for further study. For this candidate stable RNAi and overexpression (OE) lines were generated and characterized. The OE of *SlWD40* promoted ripening while its inhibition inhibited it. The co-expression networks, metabolome and transcriptome analysis indicated that *SlWD40* acted as a positive regulator of tomato ripening with the key ripening TFs such as *RIN*, *NOR*, *AP2a*, and *SlWRKYs*. These results are discussed within the context of their implications regarding fruit ripening as well as with respect to the utility of genomic information in filling our knowledge gaps in important biological processes.

## Results

### Integrating comparative transcriptomics and eQTL mapping to mine for genes involved in tomato ripening

**Figure 1:**
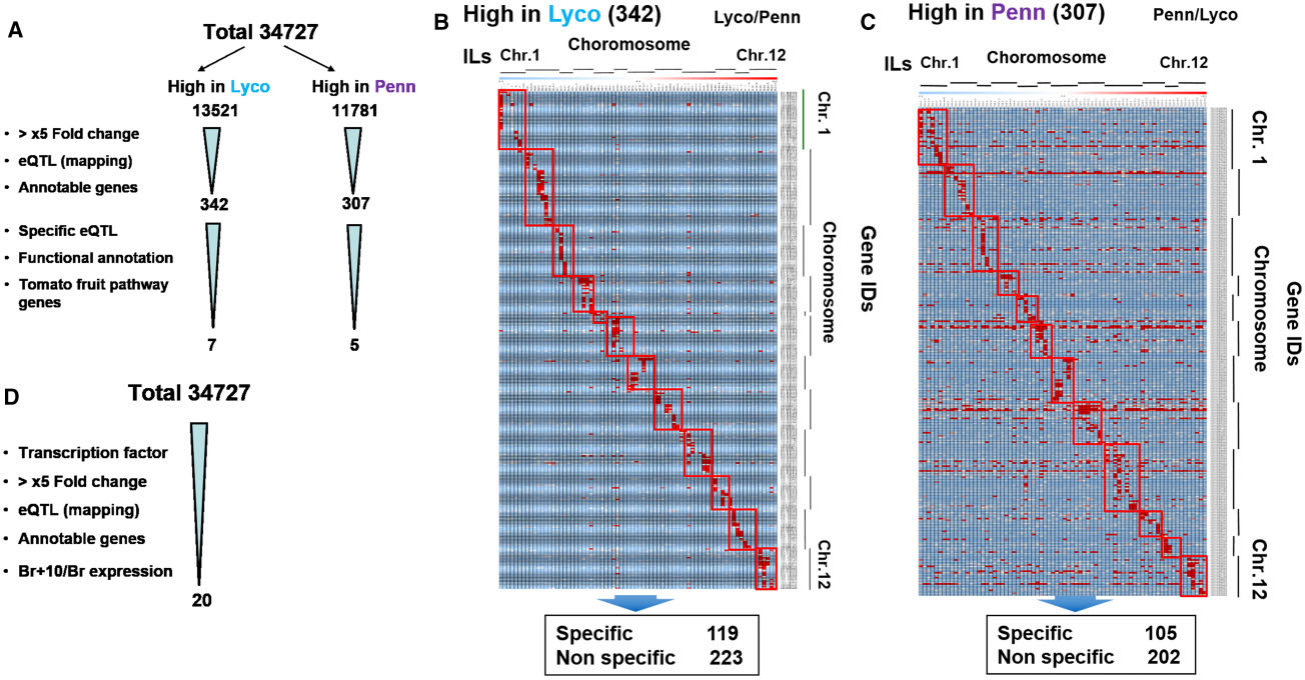
Candidate gene filtration by integrating comparative transcriptomics and eQTL mapping. A, Pipelines for candidate gene filtration of eQTL approach. Filters are shown in bullet points. B, Heat map of relative expression level of filtered candidates. Lyco/Penn, Genes were sorted by ratio of expression value for *S. lycopersicum* and *S. pennellii*. ILs are arranged as per the number of chromosome (*X*-axis). Genes are arranged according to their Gene IDs (*Y*-axis). Regions of red or blue indicate that the gene expression is increased or decreased, respectively, over that of M82. Chr, chromosome. C, Heat map of relative expression level of filtered candidates. Penn/Lyco, Genes were sorted by the ratio of expression value for *S. pennellii* and *S. lycopersicum*. ILs are arranged as per the number of chromosome (*X*-axis). Genes are arranged according to their Gene IDs (*Y*-axis). Regions of red or blue indicate that the gene expression is increased or decreased, respectively, over that of M82. D, Pipelines for candidate gene filtration of TFs approach. Filters are shown in bullet points. Br, Break.

Our earlier work described a high-quality genome assembly of the parents of the *Solanum pennellii* IL population as well as identifying candidate genes involved in salt as well as drought stress tolerance (Bolger et al., 2014). Surprisingly, the open-reading frame sequence of most well-characterized ripening-related genes is identical between *S. pennellii* and S. *lycopersicum*. We therefore thought to try a comparative transcriptomics approach of the *S. pennellii* IL population since studies on fruit gene expression of a subset of the ILs has proven highly informative (Baxter et al., 2005; Alseekh et al., 2015) as well as in leaves (Chitwood et al., 2013). For this purpose, as an initial approach, transcriptome data for M82 and *S. pennellii* mature fruits were sorted as follows: the total 34,727 genes in transcriptome sorted into two different data sets named as Lyco and Penn (Figure 1A) based on the ratio of their expression values. The Lyco data set contained genes that are highly expressed in M82 (13,521), while the Penn data set contained genes that are highly expressed in *S. pennellii* (11,781) (Figure 1A). For the Lyco genes which we reasoned would be more likely to harbor genes underlying the “red” ripe phenotype of cultivated tomatoes, around 300 candidates could be narrowed down by using three independent filters. Firstly, we chose to focus on genes for which expression was at least five times higher in *S. lycopersicum* with respect to *S. pennellii*. Secondly, based on the transcriptome profiling of red ripe fruit from *S. lycopersicum* (M82) parent and a set of lines with distinct introgressed *S. pennellii* segments (http://ted.bti.cornell.edu/cgi-bin/TFGD/array_data/home.cgi), large numbers of specific eQTL and nonspecific eQTL have been identified as the former definition that specific eQTL candidates are the candidates whose expression are sharply (exponentially) increased or decreased in its located IL compared with other 75 ILs while nonspecific eQTL candidates’ expression does not show such specificity and have expression in all ILs. With the specific eQTL and nonspecific eQTL information, the candidates of Lyco data set were classified into 119 specific and 223 nonspecific eQTLs (Figure 1B). For the Penn data set, around 105 specific and 202 nonspecific candidates were classified (Figure 1C). Here, the high number of nonspecific eQTL is attributed to the epistatic interactions between *S. pennellii* alleles and M82 alleles or, alternatively, the presence of a large number of trans-QTL as previously reported for leaf expression analysis (Chitwood et al., 2013) and fruit enzyme abundance analysis (Steinhauser et al., 2010). Previous studies have indicated that the candidates whose functional categories belong to transcription regulators, oxidase and cytochrome P450 may be involved in regulating tomato fruit ripening and secondary metabolism; therefore, we chose seven candidate genes that were of these three functional categories among the specific eQTL candidates.

In a parallel approach, given that TFs act as important regulators in fruit ripening, we also adopted a TF-centric approach (Figure 1D). From the total of 34,727 genes, candidates annotated as TFs and displaying more than five times higher expression in *S. lycopersicum* were selected. Next, eQTL mapping thinned the list to 127 candidates which were then arranged with respect to the ratio of their expression in Breaker + 10 to that in Breaker stage. Based on putative ortholog information (*Arabidopsis thaliana* and *S. lycopersicum*) and literature survey concerning their putative functions, a final set of 20 candidates was selected. Finally, on the basis of tissue specific expression and the *S. lycopersicum* to *S. pennellii* expression ratio, the 7 candidates from eQTL approach and 20 candidates from TF approach were narrowed down to the 16 potential candidates described in Supplemental Data Set S1.

### VIGS analysis of candidate genes

**Figure 2:**
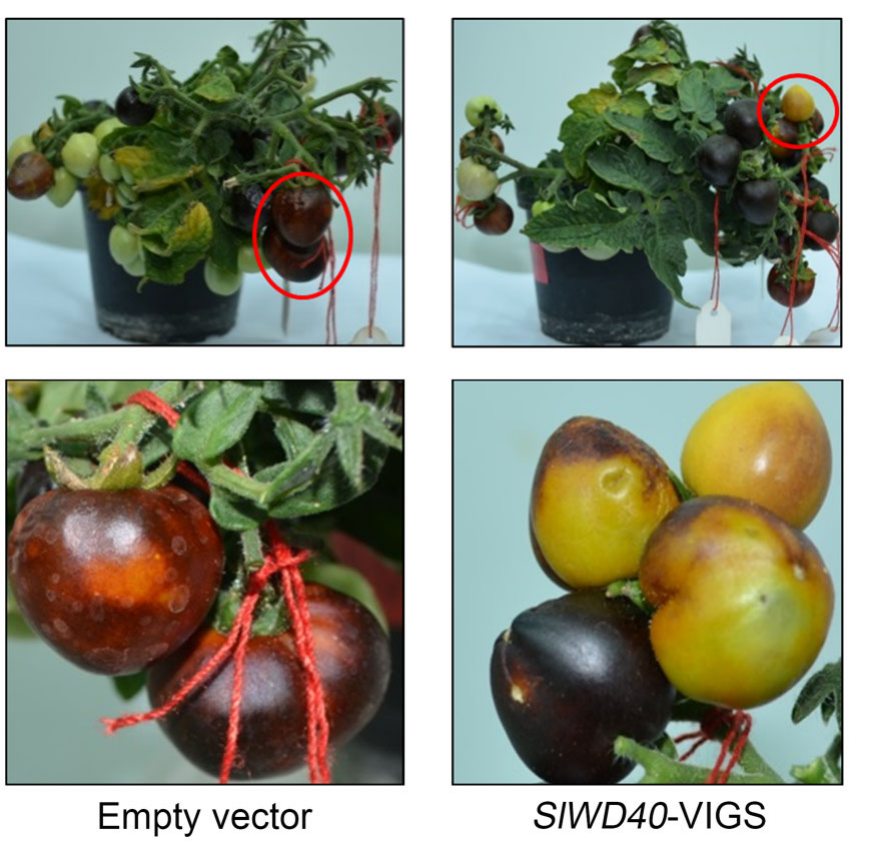
VIGS of empty vector (silencing of *Del/Ros1*) and *SlWD40* in Microtom *Del/Ros1* fruit.

To provide preliminary analysis of the function of the candidate genes in tomato ripening, we carried out VIGS experiment using purple Microtom cv. tomato fruit which accumulate high amount of anthocyanin resulting from the introduction of *Del/Ros1* petunia (*Petunia hybrida*) TFs (Orzaez et al., 2009). Partial fragments of the 16 candidate genes were cloned into pTRV2-Ros/Del/GW vector. Around 10–15 fruits per plant were infected with agrobacterium carrying the respective VIGS vector. After silencing *Del/Ros1* (empty vector) in Microtom *Del/Ros1* fruits, there was depletion in purple pigments but not in lycopene content due to the silenced part accumulating less purple anthocyanin pigments and thereby being easy to discriminate from nonsilenced (purple pigment rich) tissues (Figure 2). Phenotypes were scored visually after 15 d of infection for all the 16 validated candidates (Supplemental Data Set S1).

Red color of western tomato cultivars represents the accumulation of lycopene, which is an important indicator of tomato ripening. VIGS for the structural genes encoded by Solyc01g094080 and Solyc03g095900 displayed a red phenotype, indicating that these genes are not associated with lycopene biosynthesis or the pathways that fuel it. However, as the TFs regulating ripening are generally reported to hinder carotenoid biosynthesis, the yellowish phenotypes of Solyc11g010710 (ethylene response factors, ERF TF) and Solyc07g052700 (MADS-box TF, AGL66) VIGS fruits indicate that they may function as a ripening regulators in line with former studies that implicated *SlERFs* and MADS-box TFs in tomato fruit ripening (Wang et al., 2014; Liu et al., 2016; Supplemental Data Set S1 and Supplemental Figure S1). Interestingly, the VIGS fruits of a transcription regulator, *SlWD40* (Solyc04g005020) also exhibited a yellowish phenotype. Given that ERF and MADS box family TFs are already well-known to be involved in tomato fruit ripening and that *SlWD40* was identified as a downstream target gene of RIN (Fujisawa et al., 2013), we selected *SlWD40* for in-depth analysis here (Figure 2).

### Co-expression network and VIGS of *SlWD40* confirmed its role in tomato fruit ripening

In order to analyze the function of *SlWD40* on fruit ripening, we initially identified its potential regulators following cis-regulatory element analysis of the promoter of *SlWD40*. This analysis indicated that the promoter contained several ethylene (AP2, B3, EIN3, and EIL) and ripening-related elements (C2H2, MADS, NF-YB, NF-YA, and NF-YC) in the 1-kb promoter region, which indicated that it may well be induced by the ripening and ethylene burst (Supplemental Figure S2). Moreover, the evaluation of publicly available expression data with tissue-specific expression analysis of *SlWD40* confirmed the hypothesis that *SlWD40* was only slightly expressed in the leaf, bud, flower, root, and young fruit but that its expression increases exponentially following mature green stage (Tomato Genome Consortium, 2012). Intriguingly, its expression in different cell types of the tomato fruit revealed that it is highly similar to that of the other known ripening regulators, such as *RIN* and *NOR* (Shinozaki et al., 2018).

**Figure 3:**
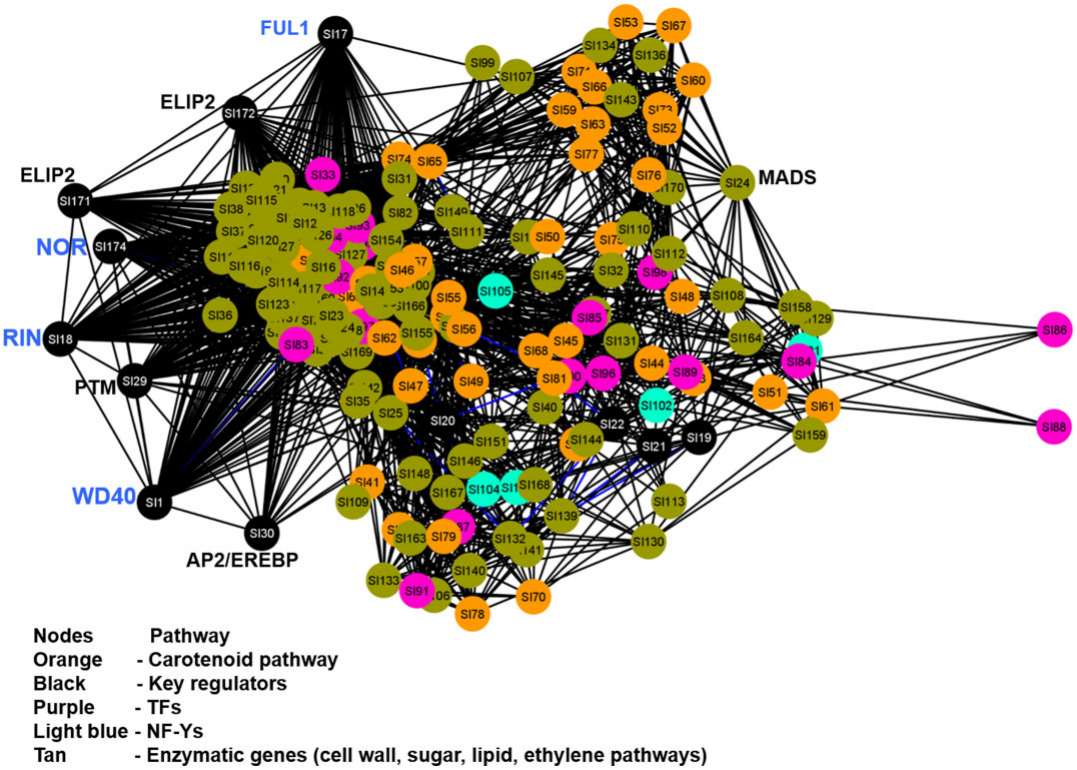
Co-expression network of *SlWD40* with tomato ripening pathway-specific genes. Well-characterized key regulators such as RIN (ripening inhibitor), NOR (nonripening), and FUL1 (FRUITFUL1) (labeled in blue) strongly co-expressed with *SlWD40*. ELIP, early light induced protein; NF-Y, nuclear factor Y; MADS, MADS domain protein.

Moreover, given that assembly of co-expression networks is an efficient method to identify the important interactions and relationship among different genes (Mutwil et al., 2011), available transcriptome data of different organ and fruit development stages were used to construct tomato co-expression network (Figure 3 and Supplemental Table S1; Tomato Genome Consortium, 2012). The co-expression sub-network containing *SlWD40* included 171 structure genes/regulators, which are involved in chlorophyll and carotenoid metabolism as well as tomato fruit ripening and cell wall metabolic pathways. Among the 171 genes, a total of 62 genes exhibited high co-expression phenotype (|Co-expression Coefficients| >0.6, *P* < 0.05) with *SlWD40* (Supplemental Table S1). Consistent with the results of cis-regulatory element analysis, three MADS TFs, including *RIN*, two AP2s TFs, and one ARF TF were significantly positively co-expressed with *SlWD40*. Moreover, another vital ripening-related TF, *NOR*, exhibited a co-expression coefficient of 0.86 with *SlWD40* (Supplemental Table S1). Besides the master ripening-related TFs, *SlWD40* also highly co-expressed with key carotenoid-related genes (*PSY1*), as well as ethylene (*ACS4*) and abscisic acid (*NCED3*) biosynthesis genes and cell wall modification genes (*PL* and *PMEI*) (Supplemental Table S1). All of these results indicate that *SlWD40* may act in concert with the better characterized ripening TFs to regulate the ripening processes, including those dependent on changes in pigmentation, hormone levels, and signaling and cell wall modification.

### *SlWD40* affects the tomato fruit transcriptome

To confirm the accuracy of our approach and to assess in detail the function of *SlWD40* in the tomato ripening process, we chose the fruit specific patatin B33 promoter which has been widely used for fruit specific expression to carry out the stable transformation (Rocha-Sosa et al., 1989; Vallarino et al., 2020). T0 transformants of RNAi and OE lines were characterized by NPT-II-specific polymerase chain reaction (PCR). Real-time quantitative polymerase chain reaction (RT-qPCR) was also carried out using fruit samples from promising T0 transgenics to select high OE and knockdown lines to raise T1 generation (Supplemental Figure S3). Fruits from all generations were analyzed and phenotype was stable over T0 and T1 generations. Before the T1 generation plant transplant to soil, we also used the NPT-II-specific PCR to confirm that the plants are transgenic. Based on the expression of *SlWD40*, two independent lines of RNAi (RNAi-1 and -2 lines) and OE (OE-1 and -2 lines) were chosen for subsequent experiments (Supplemental Figure S3).

**Figure 4:**
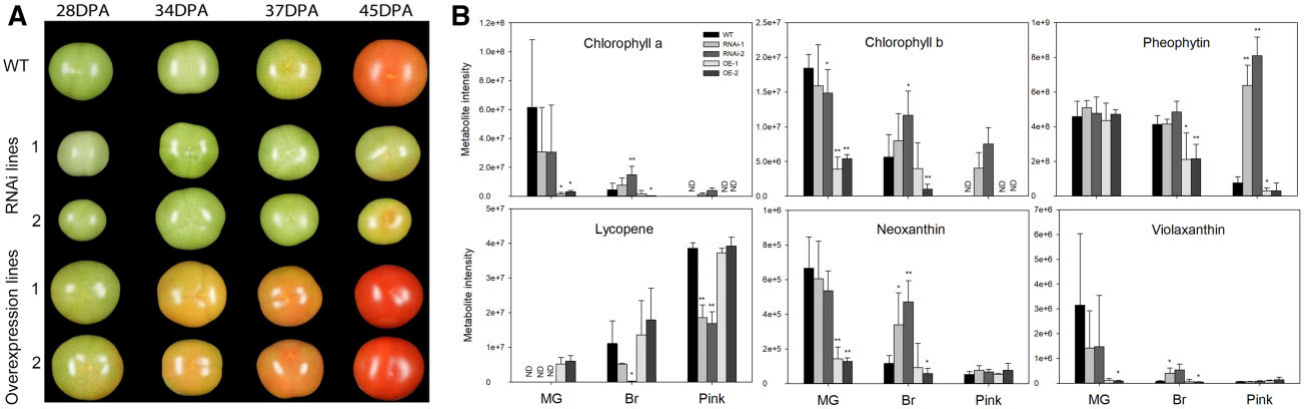
Photographs and pigments content of WT and T1 generation RNAi (lines 1 and 2) and OE (lines 1 and 2) lines at 28, 34 (MG), 37 (Br), and 45 (Pink) DPA fruits. A, Photographs of WT and transgenic *SlWD40* fruits. Images were digitally extracted for comparison. B, Chlorophylls and carotenoids of WT and transgenic *SlWD40* fruits. The values in each column are the mean of at least three biological replicates. Error bars indicate sd. The asterisks indicate statistically significant differences determined by the Student’s *t* test (two-tail): *P <* *0.05; **P <* *0.01. ND, not detected.

To analyze fruit phenotype at the identical stage, fruits of each genotype were labeled upon anthesis and harvested for phenotyping, transcriptome, and metabolite profiling at mature green (MG, 34 DPA), breaker (Br, 37DPA), and pink (Pink, 45 DPA) stages of the wild type (WT). As seen in Figure 4, the development and ripening process were substantially hindered in the RNAi fruit while the ripening process was significantly accelerated in comparison to the OE lines. Moreover, especially at the Br stage of WT fruits, the size of RNAi fruits was significantly smaller than that of the OE and WT lines and the RNAi fruits were still at the mature green stage while the OE fruit were almost at the pink stage. The contents of chlorophylls and carotenoids, some of the most important parameters of fruit ripening, also confirmed the positive function of *SlWD40* on tomato ripening process: The degradation of chlorophylls and synthesis of the predominant carotenoid, lycopene, were significantly hindered in RNAi fruits but accelerated in the OE fruits (Figure 4B).

**Figure 5:**
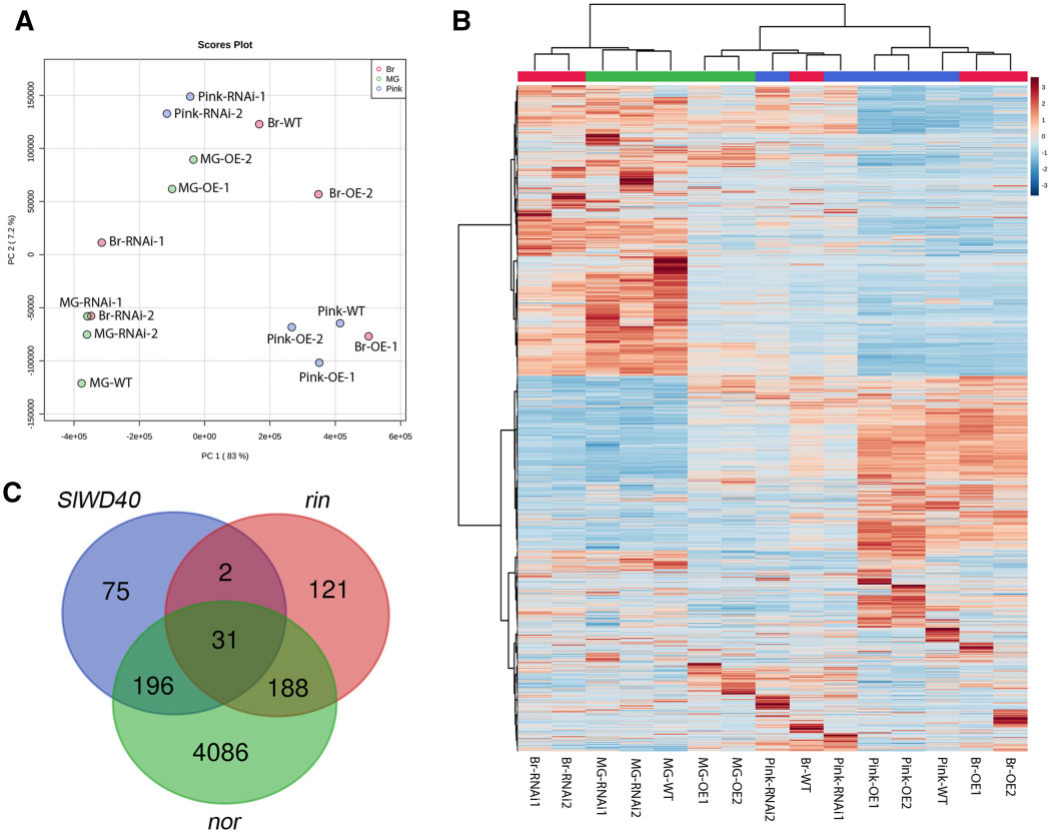
The effect of *SlWD40* on fruit transcriptome. A, PCA of transcriptome of different genotypes in the three fruit stages. PC, principal component. B, Heat map of transcriptome profiles of pericarp tissues of T1 *SlWD40* RNAi, OE lines, and WT. *X*-axis indicates different transgenic lines along with different fruit ripening stages. The hierarchical clustering based on transcriptome indicated that the development of RNAi samples is delayed especially on Br stage which are cluster with MG stage samples. C, Venn diagram showing the overlap DEGs between *SlWD40* transgenic fruits, *rin* and *nor* mutant fruits.

In order to estimate the effect of *SlWD40* on the global difference of gene expression during the different fruit developmental stages, we additionally analyzed the differentially expressed genes (DEGs) among the RNAi, OE lines, and WT fruit at MG, Br, and Pink stages. For this purpose, we used FPKM (fragments per kilobase per million mapped fragments) and identified genes with |log_2_ (fold change) | ≥ 1 and false discovery rate (FDR) (corrected *P* value) < 0.05 (Supplemental Table S2). Firstly, we checked the *SlWD40* expression among the different genotypes at the MG stage. Given the low expression level of *SlWD40* of WT fruit at MG stage, RNAi fruit did not exhibit significantly different expression from WT fruit. However, as the ripening process was initiated, the expression of *SlWD40* was significantly induced and its expression was remarkably lower in the RNAi fruit compared with that of WT fruit at Br and Pink stages without affecting the expression of other WD40 family genes (Supplemental Table S2). In the OE fruits, *SlWD40* expression was 5.64- and 5.71-fold higher than that of WT fruit at the MG stage. That said owing to the massive induction of the endogenous *SlWD40* expression, the OE effect of B33 promoter was concealed and *SlWD40* expression was not significantly different between the OE and WT fruit at the Br and Pink stages (Supplemental Table S2). These results were further confirmed by the principal component analysis (PCA) and cluster analysis based on the transcriptome data of different samples. RNAi samples were closely grouped with WT at MG stage but substantially separated samples at Br and Pink stages. Conversely, OE samples were clustered with WT sample especially at the Pink stage and subsequently separated from the WT sample at the MG stage (Figure 5, A and B). In order to further mine the important DEGs under the effect of *SlWD40*, we further analyzed the overlapping DEGs of OE-WT fruit at MG stage and RNAi-WT fruits at Br and Pink stage. The results indicate that 244 genes were stably downregulated in the OE-MG and upregulated in the RNAi Br and Pink stages, while 60 genes were stably upregulated in the OE-MG and downregulated in the RNAi Br and Pink stages (Supplemental Table S3). To further mine the functional categorization of DEGs, AgriGO v2.0 analysis tools (http://bioinfo.cau.edu.cn/agriGO/) by singular enrichment analysis has been used based on the conserved DEGs (Wang et al., 2017; Supplemental Table S4). Among the GO terms included in the “Molecular Function” category of the DEGs upregulated in the OE-MG and downregulated in the RNAi Br and Pink stages, the pathways that affected the lysis and enzyme activity, such as lyase activity (FDR = 0.0014), oxidoreductase activity (FDR = 0.011), and monooxygenase activity (FDR = 0.013), were enriched. Moreover, in the “Biological Process” category of DEGs downregulated in the OE-MG and upregulated in the RNAi Br and Pink stages, several cell-wall-related pathways were significantly enriched (Supplemental Table S4). Interestingly, in the *SlWD40*-OE lines, the expression of *SlWRKY75* (Solyc05g015850.4.1), *SlWRKY37* (Solyc02g021680.3.1), *SlWRKY23* (Solyc01g079260.4.1), *SlWRKY30* (Solyc07g056280.3.1), *SlWRKY6* (Solyc02g080890.3.1), *SlWRKY17* (Solyc07g051840.4.1), *SlWRKY31* (Solyc06g066370.4.1), and *SlWRKY79* (Solyc02g072190.4.1) was increased by 5.7-, 3.4-, 2.6-, 2.6-, 2.4-, 2.2-, 2.2-, and 1.8-fold, respectively. Moreover, since fruit size of *SlWD40*-RNAi fruits was smaller and IAA content directly affects organ size, we found that the expression of *SlGH3-2* (gene regulating auxin homeostasis) was increased by three-fold in OE lines while the level of the same gene was decreased in RNAi lines by three- to six-fold.

**Figure 6:**
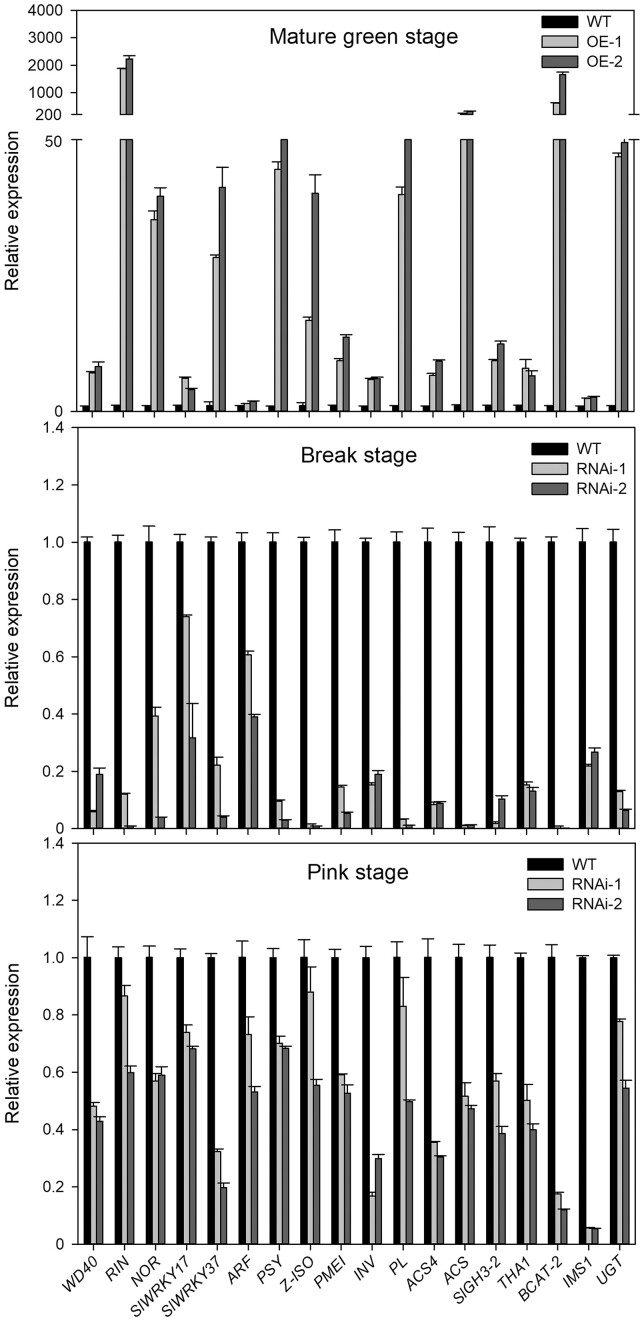
The different expression of *SlWD40* and other 17 ripening-related genes of T1 transformants of RNAi and OE lines compared with WT fruits. The values in each column are the mean of three biological replicates. Error bars indicate sd.

Additionally, given that *SlWD40* was strongly co-expressed with *RIN* and *NOR*, the conserved DEGs of *rin*, *nor* mutants, and *SlWD40* transgenic fruits were analyzed and 31 genes were found as conserved DEGs across the genotypes (Figure 5C and Supplemental Table S5; Fujisawa et al., 2012; Gao et al., 2020). Among the 31 genes, several important ripening-related genes, such as *ACS4* for ethylene biosynthesis, *PSY* and *Z-ISO* for carotenoid biosynthesis, *PL* and *PMEI* for cell wall modification, *INV* for sugar metabolism, and *branched chain amino transferase 2* (*BCAT2*) and *THA1* for amino acid metabolism, were significantly downregulated in the *SlWD40*-RNAi, *rin*, and *nor* fruits and upregulated in the *SlWD40*-OE fruits, which indicates that *SlWD40* may participate in some or all of the primary regulatory functions of these important regulators, such as *RIN* and *NOR*. As the transcriptome data indicated that *SlWD40* exhibited substantially changed expression in the MG stage of two OE lines and Br and Pink stage of two RNAi lines compared with that of in the WT, to further confirm the expression difference, we checked the expression of *SlWD40* and other 17 ripening-related genes such as *RIN*, *NOR*, *PSY*, and *PL* in these samples. The results indicate that all of these genes were significantly upregulated in the two OE lines at the MG stage but significantly downregulated in the two RNAi lines at Br and Pink stages, which further confirm the positive function of *SlWD40* on tomato ripening with the strongly co-expressed TFs (Figure 6).

### Metabolome analysis of *SlWD40* transgenic fruits

**Figure 7:**
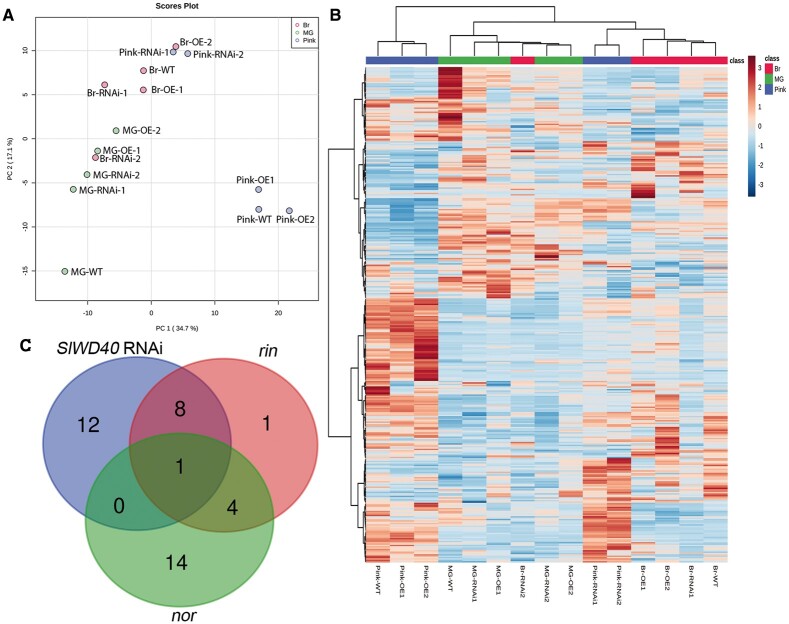
The effect of *SlWD40* on fruit metabolism. A, PCA of metabolite levels of different genotypes in the three fruit stages. PC, principal component. B, Heat map of metabolite profiles of pericarp tissues of T1 *SlWD40* RNAi, OE lines, and WT. *X*-axis indicates different transgenic lines along with different fruit ripening stages. *Y*-axis indicates metabolites. The hierarchical clustering based on all metabolites indicated that the development of RNAi samples is delayed especially in the Pink stage which are cluster with Br stage samples of OE and WT. C, Venn diagram showing the overlap different primary metabolite between *SlWD40* RNAi fruits, *rin* and *nor* mutant fruits at the Pink stage.

**Figure 8:**
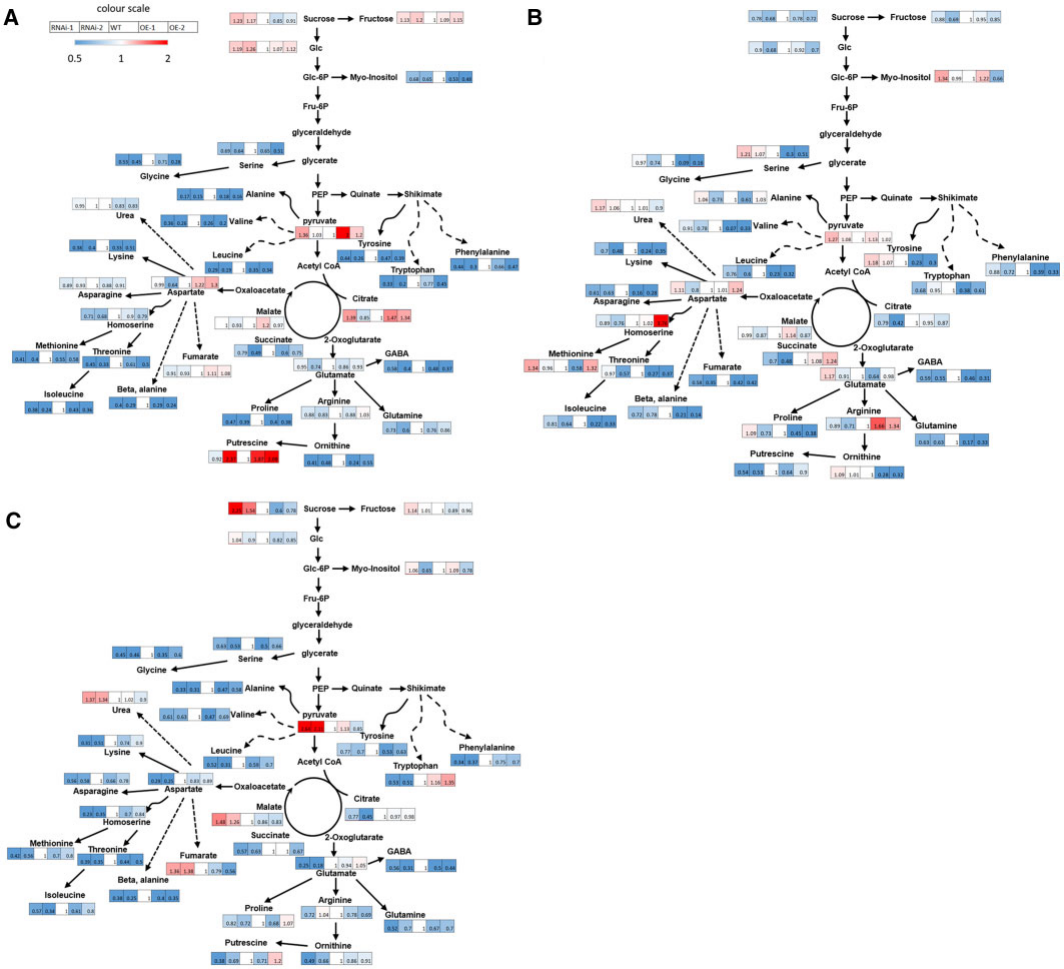
The scheme of major metabolic changes of the transgenic lines. The difference of sugar and amino acid-related metabolites between transgenic lines and WT fruit at MG (A), Br (B), and Pink (C) stage. Blue and red color depicts a decrease and increase in metabolic levels compared with the WT fruit samples, respectively.

**Figure 9:**
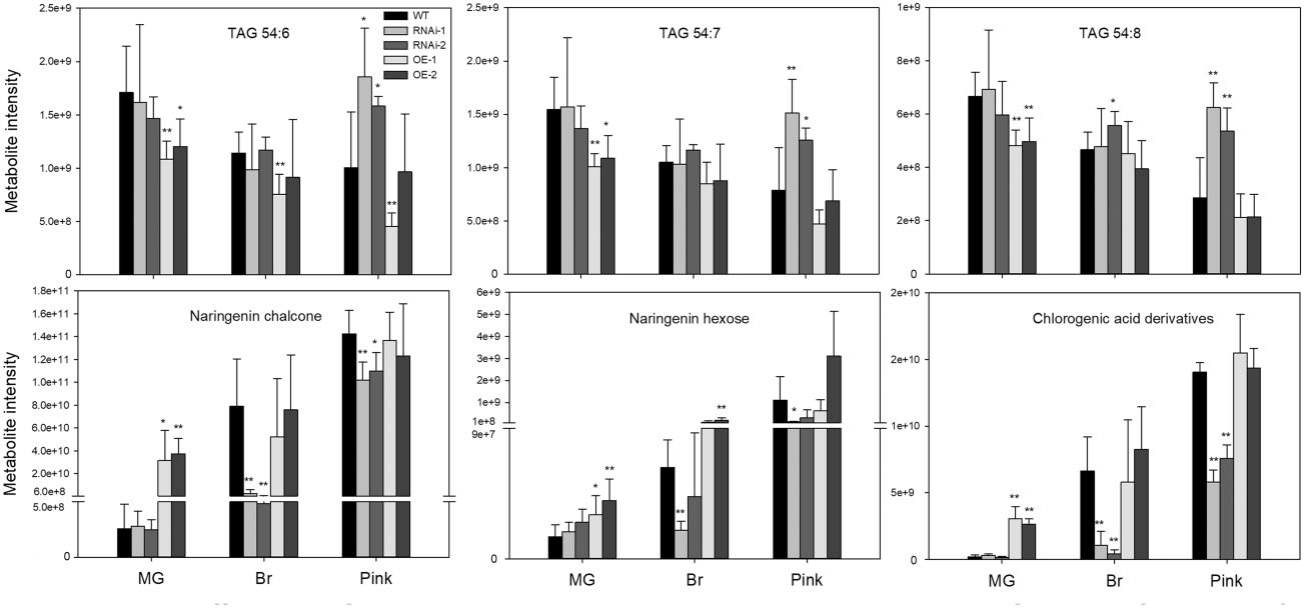
The difference of representative lipids and secondary metabolites of T1 transformants of RNAi and OE lines compared with WT fruits. The values in each column are the mean of at least three biological replicates. Error bars indicate sd. The asterisks indicate statistically significant differences determined by the Student’s *t* test (two-tail): *P <* *0.05; **P <* *0.01.

Given that metabolites are important parameters for estimating progression of the fruit ripening process (Carrari et al., 2006), we analyzed their levels in the different genotypes at a time concurrent to the MG, Br, and Pink stages of WTs. To obtain the global metabolome variation of different samples, a PCA analysis was carried out based on the primary metabolites, lipids, and secondary metabolites. The results indicate substantial differences among RNAi, OE, and WT fruits, which were particularly prominent at the Pink stage. RNAi pink fruits were closest to Br fruits and separated clearly from OE and WT fruits (Figure 7, A and B). Given that *SlWD40* was strongly co-expressed with *RIN* and *NOR*, we analyzed the overlapping differentially enriched primary metabolites between *SlWD40-*RNAi lines, *rin* and *nor* mutants at the Pink stage (Osorio et al., 2011). The results indicated that as the principal free amino acid that most contributes to the “umami” flavor of ripe tomato fruits, glutamate exhibited a conserved lower accumulation in the *SlWD40-*RNAi lines, *rin* and *nor* pink fruits (Figure 7C). In detail, given that several amino acids are increased and organic acid decreased during WT tomato ripening (Carrari et al., 2006), it is of interest that we observed that aspartic acid, glutamic acid, and tryptophan levels were significantly lower in both RNAi lines compared with those exhibited by the OE and WT pink fruits (Figure 8 and Supplemental Table S6). Moreover, nicotinic acid and glyceric acid levels were significantly higher in at least one RNAi line compared with those observed in OE and WT pink fruits (Figure 8 and Supplemental Table S6). Moreover, the former results indicated that the representative lipid components, triacylglycerols (TAGs) are significantly decreased in avocado fruit ripening (Rodriguez-Lopez et al., 2017). The same trends also have been found in the present study that the content of TAG 48:0, TAG 52:5, TAG 52:6, TAG 54:6, TAG 54:7, and TAG 54:8 was remarkably higher in the RNAi fruits than that of OE and WT pink fruits (Figure 9 and Supplemental Table S6). As the secondary metabolites (such as naringenin chalcone, naringenin hexose, and chlorogenic acid derivatives) dramatically accumulate during ripening and represent an important quality of fruit, the significantly higher and lower content of them in the OE MG fruits and RNAi pink fruits compared with that of WT fruits, respectively, further confirmed the positive influence of *SlWD40* on the fruit ripening process (Figure 9 and Supplemental Table S6).

## Discussion

We adopted a computational approach to mine key candidate genes involved in tomato fruit ripening by integrating comparative transcriptomics and eQTL analysis. In doing so, we identified 16 previously uncharacterized candidate genes for involvement in ripening. Among them, we chose to follow up on *SlWD40* since it was identified in the ChIP seq screen as a direct target gene of RIN and relatively little is known concerning its regulatory role (Fujisawa et al., 2013). Intriguingly, cross-tissue co-expression networks for *SlWD40* strongly suggest that it may act with the other important ripening regulators, such as *RIN* and *NOR* in affecting tomato fruit ripening, and the detailed analysis of DEGs and differentially abundant metabolites between *SlWD40* transgenic fruits further illuminate the important function of *SlWD40* on tomato ripening process.

### Integrating comparative transcriptomics with the eQTL approach to mine for ripening regulators

Several tomato genes with strong ripening phenotypes have been identified via mutagenesis-based breeding programs including *rin*, *nor*, *NR*, and *Cnr* (Tigchelaar, 1973; Lanahan et al., 1994; Vrebalov et al., 2002; Manning et al., 2006). Moreover, the recombinant inbred line (RILs) population is also useful to identify key loci regulating quantitative traits including fruit ripening as well as aroma, color, and disease resistance (Kimbara et al., 2018). However, since RILs usually harbor more than one introgression, the possibility of the introgressed loci having beneficial/inhibitory interactions with the genes in the genetic background renders gene function elucidation more complex in RILs. In contrast, ILs are high resolution in that they normally carry only single introgressions and as such epistatic interactions masking single gene effects are largely minimized (Ofner et al., 2016). Here, we used available transcriptome data from red ripe fruit of *S. lycopersicum* (M82) parent and a set of lines with distinct introgressed *S. pennellii* segments (http://ted.bti.cornell.edu/cgi-bin/TFGD/array_data/home.cgi) and optimized a candidate gene filtration pipeline. Moreover, on the basis of the distance between QTL and the target transcript, eQTL can either be classified as cis-eQTL (where the gene encoding the transcript resides within the QTL interval) or trans-eQTL with both types being prominent in tomato (Rockman and Kruglyak, 2009; Zhu et al., 2018). Combining the gene filtration pipeline and the comparative transcriptomics, we filtered genes responsible for the red ripe phenotype of tomato fruits (i.e. genes highly expressing only in “red” ripe fruits). Since gene duplication and subsequent functional diversification creates novel metabolic pathways and regulation, we focused on TF families that were evolved by gene duplication and already reported to be regulating tomato fruit ripening (e.g. MADS box and basic helix loop helix (bHLH) TF families) (Hileman et al., 2006; Waseem et al., 2019). Out of the 16 candidate genes, 10 were found to be generated either through tandem or block duplication (https://bioinformatics.psb.ugent.be/plaza). In the VIGS experiment, green and yellow phenotypes have been obtained for the candidates Solyc11g010710 (AP2 like) and Solyc07g052700 (MADS TF, AGL66). Our previously published work implicated AP2a in regulating tomato fruit ripening via regulation of ethylene biosynthesis and signaling (Chung et al., 2010; Karlova et al., 2011). MADS box TFs such as *SlCMB1* (Solyc04g005320), *TAGL1* (Solyc07g055920), and the canonical TF *RIN* (Solyc05g012020) have previously been reported as positive regulators of tomato fruit ripening (Zhang et al., 2018). Therefore, VIGS for AP2 like and MADS box candidates (Solyc11g010710 and Solyc07g052700) acted as positive control supporting the efficacy of our approach. Moreover, among the 16 genes, VIGS for two bHLH TFs (Solyc03g044460 and Solyc12g098620) showed green and light red phenotypes (Supplemental Figure S1). Here, light red phenotype for Solyc12g098620 is in line with the recently published work by D’Amelia et al. (2019) in which the authors reported that this bHLH TF regulates carotenoid biosynthesis. Additionally, one of the candidates, Solyc12g010950 (alcohol dehydrogenase), obtained a whitish green fruit phenotype, and another candidate, *SlWD40*, obtained a yellowish fruit phenotype (Figure 2 and Supplemental Figure S1). While the link between the alcohol dehydrogenase and ripening is currently unclear, that for *SlWD40* is not without precedence since it is a transcriptional regulator which has been linked to *rin* and *nor* in ChIP experiments but not characterized in detail in its own right.

The eQTL for *SlWD40* can be defined as a cis-eQTL since *SlWD40* is located in the region which has been introgressed by *S. pennellii* chromosomal architecture in the IL4-1 and IL4-1-1 and *SlWD40*’s expression is only dramatically lowered for IL4-1 and 4-1-1 (Supplemental Table S7). At the onset of fruit ripening process (MG stage), *SlWD40* is moderately expressed in *S. lycopersicum* (RPKM mean value 31) but negligibly expressed in *S. pennellii* (RPKM mean value 0.17) and consistent with the ripening stage, the expression of *SlWD40* was substantially induced in concert with fruit ripening. Considering the dramatically lowered *SlWD40* expression in IL4-1 and 4-1-1 and substantial difference of the red/green colored fruits of *S. lycopersicum* and *S. pennellii*, respectively, we hypothesized that *SlWD40* may participate in the *S. lycopersicum* ripening process. Furthermore, VIGS for *SlWD40* in MicroTom inhibited normal red coloration. These results demonstrate the utility of our candidate gene filtration pipeline integrating comparative transcriptomics with eQTL in identifying candidate ripening genes.

### *SlWD40* is an important regulator of tomato fruit ripening

Among the eight genes which we validated by VIGS, the information concerning the role of the WD40 family in regulating tomato ripening is the most limited. Ripening function of this gene was further confirmed by the stable OE and RNAi transformation (Figures 4 and 5). WD40 proteins contain a signature WD (Trp-Asp) dipeptide and 40 amino acids in single repeats that then fold into four-stranded anti-parallel β-propeller sheets and are highly promiscuous interactors, being both platforms for protein–DNA and protein–protein interactions (Xu and Min, 2011; Mishra et al., 2012; Chen et al., 2022). In the canonical MYB–bHLH–WD40 protein complex, WD40 acts as a recruiter and stabilizer of the MYB and bHLH protein which enhances the regulation of the complex during anthocyanin biosynthesis (Ramsay and Glover, 2005; Zhang et al., 2014). In the present study, the green phenotype of fruits of *SlWD40-*RNAi lines clearly demonstrated a positive role of *SlWD40* in tomato fruit ripening (Figure 4). Moreover, given that the global gene co-expression analysis is a powerful approach to identify the important interactions among different genes during the development of certain organs, it is important to note that the co-expression network of *SlWD40* revealed links with *RIN* and *NOR*, while transcriptome analysis indicated they shared conserved regulated genes associated with ethylene (*ACSs*) and carotenoid (*PSY* and *Z-ISO*) biosynthesis as well as cell wall degradation (*PL* and *PMEI*) and sugar (*INV*) and amino acid (*BCAT2* and *THA1*) metabolism (Supplemental Table S5). Interestingly, based on available ChIP and transcriptome data, the RIN protein can directly bind to the promoter of *SlWD40* and thereby increase its expression (Martel et al., 2011; Fujisawa et al., 2013).

Moreover, as an important TF family member involved in ethylene signal, AP2a belonging to the AP2/ERF superfamily has been shown to inhibit ethylene biosynthesis as well as positively regulate chlorophyll degradation and carotenoid biosynthesis in tomato (Wang et al., 2019). And in *Setaria italica*, SiAP2 can bind to the *SiWD40* promoter to mediate abiotic stress responses (Mishra et al., 2012). In the present study, the promoter region of *SlWD40* was found to harbor eight different AP2 TF binding sites and *AP2a* was significantly induced in *SlWD40*-OE-MG fruits while repressed in the *WD40*-RNAi-Breaker fruits (Supplemental Table S2). Additionally, it has been well-documented that several *SlWRKYs* regulate tomato fruit ripening and lycopene accumulation (Cheng et al., 2016; Wang et al., 2017). In our analysis, *SlWD40* OE resulted in upregulation (by two- to five-fold) of a broad number of *SlWRKYs* (*SlWRKY75*, *SlWRKY37*, *SlWRKY23*, *SlWRKY30*, *SlWRKY6*, *SlWRKY17*, *SlWRKY31*, and *SlWRKY79*) in mature green fruits of OE lines. Moreover, SlWRKY17, which was shown to interact with RIN, SlERF2b, and SlERF7, is strongly co-expressed with *ELIP2* and *RIN* in our analysis (Wang et al., 2017; Supplemental Table S1). All of these results indicate that *SlWD40* might act as a junction point and facilitate binding of one or more above mentioned co-expressed TFs such as *RIN*, *NOR*, *AP2a*, and *SlWRKYs* to be involved in tomato ripening process.

Besides the important ripening-related TFs, hormone signaling also plays a vital role in the ripening process. As an important regulator of auxin-ethylene homoeostasis which affects fruit ripening (Kumar et al., 2012; Sravankumar et al., 2018), the expression of *SlGH3-2* (Solyc01g107390) increased in *SlWD40*-OE lines by three-fold while decreased in RNAi lines by three- to six-fold (Figure 6 and Supplemental Table S2). Combined with the induction and repression effect of *ACS4* in the *SlWD40*-OE and -RNAi fruit, respectively, these results collectively indicate that *SlWD40* may also affect the tomato ripening process through the regulation of the ripening-related hormone homoeostasis. However, considerably further experimentation will be required in order to test this hypothesis.

## Conclusion

**Figure 10:**
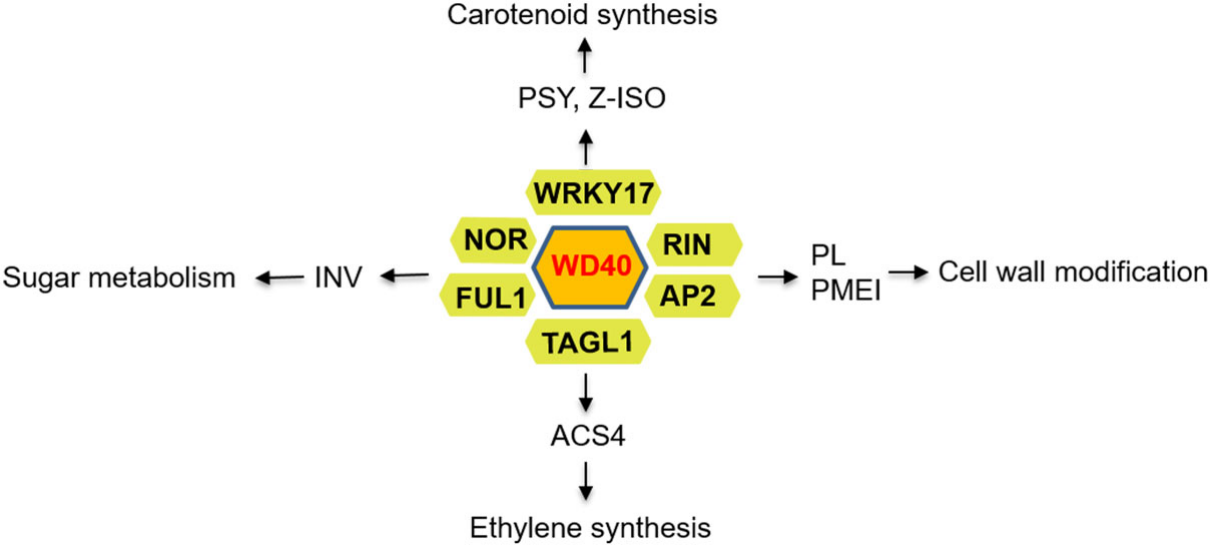
Proposed schematic overview of network of regulatory factors controlling tomato fruit ripening.

Our study aimed at better understanding the molecular mechanisms underlying tomato fruit ripening. Comparative transcriptomics of small green fruited wild species with red fruited *S. lycopersicum* alongside eQTL mapping allowed the identification of key candidate genes involved in tomato fruit ripening. Utilizing co-expression networks alongside detailed metabolome and transcriptome analysis indicated that *SlWD40* has a positive impact on tomato ripening process and suggest that it may act in concert with strongly co-expressed TFs such as *RIN*, *NOR*, *AP2a*, and *SlWRKYs* (Figure 10). Beyond these insights into ripening, we believe our study also acts as a proof-of-concept study whereby the transcriptome of phenotypically divergent wild relatives, alongside eQTL mapping, can be used to identify causal genes underlying trait variance.

## Materials and methods

### Narrowing down candidate genes involved in fruit ripening

For M82 and *S.*  *pennellii* (Penn) fruit, RNA-seq data are available (Bolger et al., 2014). Moreover, RNA-seq data for *S. pennellii* ILs fruit were also available (http://ted.bti.cornell.edu/cgi-bin/TFGD/array_data/home.cgi). Starting with the RNA-seq data for M82 and *S.*  *pennellii* fruit (Bolger et al., 2014) all 34,727 genes in the transcriptome were sorted in two ways. Firstly, the ratio of their expression value in M82 relative to that in *S.*  *pennellii* and secondly, by ratio of their expression value of *S.*  *pennellii* to that of M82 in order to get genes that are highly expressed in M82 and *S.*  *pennellii*, respectively. Candidates from these two lists were further filtered by using three different criteria namely (1) that the relative fold change (FC) in the expression was at least >5, (2) the presence of an eQTL for the gene, and (3) the presence of a functional annotation. Subsequently, these candidates were sorted into specific and nonspecific eQTL based on their expression in respective IL. Here, specific and nonspecific eQTL were defined based on the expression of a particular *S. pennellii* candidate in a specific IL or several ILs, respectively. Specific eQTL candidates were then focused and classified based on their function. For promoter analysis, the 1-kb upstream sequence from the start codon of SlWD40 was retrieved from the SGN tomato genome web browser and the cis-regulatory elements were analyzed using PlantCARE (http://bioinformatics.psb.ugentbe/webtools/plantcare/html/) and PlantPAN 2.0 (http://plantpan2.itps.ncku.edu.tw/index.html) web tools.

### *SlWD40* co-expression network construction with tomato ripening pathway genes

We listed 171 target genes involved in carotenoid biosynthesis, tomato fruit ripening, and cell wall metabolic pathways and their regulation. Some of these genes were well characterized. For all 171 genes, expression values were extracted from Tomato Genome Consortium (2012). The R script written by Contreras-Lopez et al. (2018) was used to calculate correlation values and *P* values, both positive and negative correlation values were calculated and cytoscape was used to visualize network (Shannon et al., 2003).

### Virus-induced gene silencing

Vector construction, infiltration, and fruit harvesting procedures were performed as previously described (Orzaez et al., 2006, 2009). Briefly, an approximately 300-bp fragment of the candidate gene was amplified from tomato M82 fruit cDNA using gateway compatible primers and recombined into the GATEWAY vector pDONR207 (Invitrogen, http://www.invitrogen.com/) by the BP reaction following the manufacturer’s protocol to generate an entry clone. An error-free entry vector was confirmed by sequencing and then recombined with the pTRV2-Ros/Del/GW destination vector using an LR reaction to produce the expression clones pTRV2-Ros/Del/GW-Respective Gene ID. *Agrobacterium tumefaciens* strain GV3101:pMP90 was then transformed with sequenced expression vectors by electroporation. In order to infiltrate fruit for VIGS, purple MicroTom tomato was used and agroinfiltration was performed as previously described (Alseekh et al., 2015).

### Development of OE and RNAi lines

The sequence encoding Solyc04g005020 was amplified from *S. lycopersicum* cv. Moneymaker (MM) cDNA by using gene-specific primers and inserted into the pDONR207 by attB recombination to generate entry clone. Primer sequences are provided in Supplemental Table S8. An error-free entry clone was confirmed by sequence analysis before recombination into destination vector B33BinAR for fruit-specific OE and named as B33BinAR_SlWD40. Additionally, artificial miRNA (amiRNA) cassette was designed for Solyc04g005020. For this, Solyc04g005020 cDNA sequence was used as target sequence, employing the WMD3 program (http://wmd3.weigelworld.org/cgi-bin/webapp.cgi) to design corresponding amiRs. An overlapping PCR (polymerase chain reaction) strategy was employed with in-hand precursor DNA, following the WMD3 protocol (http://wmd3.weigelworld.or/downloads/CloningofartificialmicroRNAs.pdf). The pre-amiRs obtained from overlapping PCR (using the athmir-319a backbone) were cloned into the pENTR/D-TOPO vector and the clones were confirmed by DNA sequencing. Subsequently, these sequences were cloned into B33BinAR via Asp718 and BamHI digestion and cohesive end ligation. Primer sequences are provided in Supplemental Table S8. This and other final LR plasmids were then introduced into *A. tumefaciens* strain GV2260 by electroporation and subsequently submitted for transformation into MM plants using the leaf disc transformation method (McCormick et al., 1986).

### Plant material and growth conditions

Transgenic plants for each genotype were selected on kanamycin containing MS medium (50 mg·L^-1^). SNN and MM (WT) were germinated on MS medium without kanamycin. Both transgenic lines and WT were selected and transferred to soil pot for cultivation under long-day conditions (16-h/8-h day/night cycle) at 22°C and 50% humidity, as described previously in the literature (Carrari et al., 2003). Upon anthesis, flowers were labeled with that particular date.

### Transcriptome analysis

Two biological replicate samples from two independent plants of each genotype of MG, Br, and Pink stages have been harvested. Total RNA was extracted using the NucleoSpin RNA Plant kit (Macherey-Nagel) and sent to the Novogene Company (Beijing, China) for Illumina HiSeq PE150 sequencing. The cDNA library was constructed following the manufacturer’s recommendations and then purified to remove the low-quality sequences. The clean data are available in Zenodo (https://zenodo.org/) (doi:10.5281/zenodo.5525948 and 10.5281/zenodo.5525946). The RNA seq data were analyzed using LSTrAP (Proost et al., 2017). The clean reads of each sample were aligned to the Tomato Genome version SL4.0 and Annotation ITAG4.0 (ftp://ftp.solgenomics.net/tomato_genome/annotation/ITAG4.0_release/). The DEGs between transgenic fruit and WT fruit were identified under the parameter of FC ≥ 2 and FDR < 0.05.

### Metabolic profiling

Fruit pericarp samples were harvested, immediately frozen in liquid nitrogen, and stored at −80°C until further analysis. Samples were then powdered by using retsch mill at 30 L·s^-1^, for 30 s. Extraction of pigments, primary metabolites, lipid, and secondary metabolites was performed as described previously (Salem et al., 2016). In brief, 500 µL of the upper lipid and pigments containing phase was dried in a SpeedVac concentrator and resuspended in 250 µL acetonitrile: 2-propanol (7:3, v/v) solution. Two microliters of the solution were analyzed by the Waters Acquity ultra-performance LC system coupled with Fourier transform MS in positive ionization mode. Moreover, 150 and 300 μL of the polar phase were dried in a centrifugal vacuum concentrator for primary and secondary metabolite profiling. The primary metabolite pellet was resuspended in 40 μL of methoxyaminhydrochloride (20 mg·mL^-1^ in pyridine) and derivatized for 2 h at 37°C. Afterward, 70 μL of *N*-methyl-*N*-[trimethylsilyl] trifluoroacetamide was added containing 20 μL·mL^-1^ fatty acid methyl esters mixture as retention time standards. The mixture was incubated for 30 min at 37°C at 400 rpm. A volume of 1 μL of this solution was used for injection. The gas chromatography–mass spectroscopy system comprised a CTC CombiPAL autosampler, an Agilent 6890N gas chromatograph, and a LECO Pegasus III time of flight mass spectrometry (TOF-MS) running in EI+ mode. The secondary metabolite pellet was resuspended in 200-µL 50% (v/v) methanol in water and 2 µL was injected on RP high strength silica T3 C_18_ column using a Waters Acquity UPLC system. The analysis workflow included peak detection, retention time alignment, and removal of chemical noise following the method of Salem et al. (2016). For metabolites and transcriptome data processing, the PCA and heat map analysis were performed by MetaboAnalyst 5.0 (https://www.metaboanalyst.ca/).

### RT-qPCR analysis

Total RNA was extracted from fruit using TRIzol reagent (Invitrogen, Waltham, MA, USA). And the first-strand cDNA synthesis was carried out as the manufacturer’s instructions of PrimeScript RT Reagent Kit with gDNA Eraser (Takara, Shiga, Japan). RT-qPCR was analyzed on an ABI Prism 7900 HT real-time PCR system (Applied Biosystems/Life Technologies, Darmstadt, Germany) in 384-well PCR plates. The RT-qPCR data were analyzed using the 2^−ΔΔCt^ analysis method according to Bustin et al. (2009) and all primers are listed in Supplemental Table S8.

### Statistical analysis

Student’s paired *t* test was performed to assess whether the differences between different genotypes were statistically significant. The asterisks indicate statistically significant differences determined by the Student’s *t* test (two-tail): *P <* *0.05; **P <* *0.01.

## Accession numbers

Sequence data from this article can be found in the GenBank/EMBL data libraries under accession numbers *SlWD40*, Solyc04g005020.

## Supplemental data

The following materials are available in the online version of this article.

**Supplemental Figure S1.** VIGS phenotype of candidate genes.

**Supplemental Figure S2.** Promoter analysis of *SlWD40* for the presence of ethylene (C2H2, AP2, EIN), auxin, and MADS-box binding-related cis-regulatory elements.

**Supplemental Figure S3.** Genotyping of the *SlWD40* of T0 transformants.

**Supplemental Table S1.** Co-expression network of *SlWD40* with tomato ripening pathway specific genes.

**Supplemental Table S2.** Transcriptome profiling of *SlWD40* transgenic fruits.

**Supplemental Table S3.** The overlapped DEGs of *SlWD40* OE and RNAi fruit.

**Supplemental Table S4.** Functional categorization of DEGs of *SlWD40.*

**Supplemental Table S5.** The overlap DEGs of *SlWD40*, *rin*, and *nor* mutants.

**Supplemental Table S6.** Metabolite profiling of *SlWD40* transgenic fruits.

**Supplemental Table S7.** Expression of *SlWD40* in ILs.

**Supplemental Table S8.** Primer sequences used in this study.

**Supplemental Data Set S1.** Finalized potential candidates from both eQTL and TF approaches and their VIGS phenotypes.

## Supplementary Material

kiac200_Supplementary_Data

## Notes

S.A. and A.R.F. supervised the experiments. F.Z., S.S.J., T.T., and J.M.L. performed the experiments. F.Z., S.S.J., and A.R.F. wrote the manuscript. J.J.G. and Y.C. revised the manuscript. All the authors read and approved the final manuscript.

The author responsible for distribution of materials integral to the findings presented in this article in accordance with the policy described in the Instructions for Authors (https://academic.oup.com/plphys/pages/General-Instructions) is: Alisdair R. Fernie (fernie@mpimp-golm.mpg.de).

## References

[kiac200-B1] Alseekh S , Ofner I, Pleban T, Tripodi P, Di Dato F, Cammareri M, Mohammad A, Grandillo S, Fernie AR, Zamir D (2013) Resolution by recombination: breaking up *Solanum pennellii* introgressions. Trends Plant Sci 18: 536–538 24029406 10.1016/j.tplants.2013.08.003

[kiac200-B2] Alseekh S , Tohge T, Wendenberg R, Scossa F, Omranian N, Li J, Kleessen S, Giavalisco P, Pleban T, Mueller-Roeber B, et al (2015) Identification and mode of inheritance of quantitative trait loci for secondary metabolite abundance in tomato. Plant Cell 27: 485–512 25770107 10.1105/tpc.114.132266 PMC4558650

[kiac200-B3] Ballester AR , Molthoff J, de Vos R, Hekkert B, Orzaez D, Fernandez-Moreno JP, Tripodi P, Grandillo S, Martin C, Heldens J, et al (2010) Biochemical and molecular analysis of pink tomatoes: deregulated expression of the gene encoding transcription factor SlMYB12 leads to pink tomato fruit color. Plant Physiol 152: 71–84 19906891 10.1104/pp.109.147322 PMC2799347

[kiac200-B4] Baranwal VK , Negi N, Khurana P (2021) Comparative transcriptomics of leaves of five mulberry accessions and cataloguing structural and expression variants for future prospects. PLoS ONE 16: e0252246 34260613 10.1371/journal.pone.0252246 PMC8279327

[kiac200-B5] Bartley GE , Scolnik PA (1993) cDNA cloning, expression during development, and genome mapping of PSY2, a second tomato gene encoding phytoene synthase. J Biol Chem 268: 25718–25721 8245008

[kiac200-B6] Batyrshina ZS , Yaakov B, Shavit R, Singh A, Tzin V (2020) Comparative transcriptomic and metabolic analysis of wild and domesticated wheat genotypes reveals differences in chemical and physical defense responses against aphids. BMC Plant Biol 20: 19 31931716 10.1186/s12870-019-2214-z PMC6958765

[kiac200-B7] Baxter CJ , Carrari F, Bauke A, Overy S, Hill SA, Quick PW, Fernie AR, Sweetlove LJ (2005) Fruit carbohydrate metabolism in an introgression line of tomato with increased fruit soluble solids. Plant Cell Physiol 46: 425–437 15695458 10.1093/pcp/pci040

[kiac200-B8] Bird CR , Ray JA, Fletcher JD, Boniwell JM, Bird AS, Teulieres C, Blain I, Bramley PM, Schuch W (1991) Using antisense RNA to study gene-function—inhibition of carotenoid biosynthesis in transgenic tomatoes. Bio-Technology 9: 635–639

[kiac200-B9] Bolger A , Scossa F, Bolger ME, Lanz C, Maumus F, Tohge T, Quesneville H, Alseekh S, Sorensen I, Lichtenstein G, et al (2014) The genome of the stress-tolerant wild tomato species *Solanum pennellii*. Nat Genet 46: 1034–1038 25064008 10.1038/ng.3046 PMC7036041

[kiac200-B10] Breschi A , Gingeras TR, Guigo R (2017) Comparative transcriptomics in human and mouse. Nat Rev Genet 18: 425–440 28479595 10.1038/nrg.2017.19 PMC6413734

[kiac200-B11] Bustin SA , Benes V, Garson JA, Hellemans J, Huggett J, Kubista M, Mueller R, Nolan T, Pfaffl MW, Shipley GL, et al (2009) The MIQE guidelines: minimum information for publication of quantitative real-time PCR experiments. Clin Chem 55: 611–622 19246619 10.1373/clinchem.2008.112797

[kiac200-B12] Carrari F , Baxter C, Usadel B, Urbanczyk-Wochniak E, Zanor MI, Nunes-Nesi A, Nikiforova V, Centero D, Ratzka A, Pauly M, et al (2006) Integrated analysis of metabolite and transcript levels reveals the metabolic shifts that underlie tomato fruit development and highlight regulatory aspects of metabolic network behavior. Plant Physiol 142: 1380–1396 17071647 10.1104/pp.106.088534 PMC1676044

[kiac200-B13] Carrari F , Fernie AR (2006) Metabolic regulation underlying tomato fruit development. J Exp Bot 57: 1883–1897 16449380 10.1093/jxb/erj020

[kiac200-B14] Carrari F , Nunes-Nesi A, Gibon Y, Lytovchenko A, Loureiro ME, Fernie AR (2003) Reduced expression of aconitase results in an enhanced rate of photosynthesis and marked shifts in carbon partitioning in illuminated leaves of wild species tomato. Plant Physiol 133: 1322–1335 14551334 10.1104/pp.103.026716 PMC281627

[kiac200-B15] Cazzonelli CI , Pogson BJ (2010) Source to sink: regulation of carotenoid biosynthesis in plants. Trends Plant Sci 15: 266–274 20303820 10.1016/j.tplants.2010.02.003

[kiac200-B16] Centeno DC , Osorio S, Nunes-Nesi A, Bertolo ALF, Carneiro RT, Araújo WL, Steinhauser M-C, Michalska J, Rohrmann J, Geigenberger P, et al (2011) Malate plays a crucial role in starch metabolism, ripening, and soluble solid content of tomato fruit and affects postharvest softening. Plant Cell 23: 162–184 21239646 10.1105/tpc.109.072231 PMC3051241

[kiac200-B17] Chang YM , Lin HH, Liu WY, Yu CP, Chen HJ, Wartini PP, Kao YY, Wu YH, Lin JJ, Lu MJ, et al (2019) Comparative transcriptomics method to infer gene coexpression networks and its applications to maize and rice leaf transcriptomes. Proc Natl Acad Sci USA 116: 3091–3099 30718437 10.1073/pnas.1817621116 PMC6386681

[kiac200-B18] Chen L , Li W, Li Y, Feng X, Du K, Wang G, Zhao L (2019) Identified trans-splicing of Y*ELLOW-FRUITED TOMATO 2* encoding the PHYTOENE SYNTHASE 1 protein alters fruit color by map-based cloning, functional complementation and RACE. Plant Mol Biol 100: 647–658 31154655 10.1007/s11103-019-00886-y

[kiac200-B19] Chen W , Chen L, Zhang X, Yang N, Guo J, Wang M, Ji S, Zhao X, Yin P, Cai L, et al (2022) Convergent selection of a WD40 protein that enhances grain yield in maize and rice. Science 375: eabg7985 35324310 10.1126/science.abg7985

[kiac200-B20] Cheng Y , Ahammed GJ, Yu J, Yao Z, Ruan M, Ye Q, Li Z, Wang R, Feng K, Zhou G, et al (2016) Putative WRKYs associated with regulation of fruit ripening revealed by detailed expression analysis of the WRKY gene family in pepper. Sci Rep 6: 39000 27991526 10.1038/srep39000 PMC5171846

[kiac200-B21] Chitwood DH , Kumar R, Headland LR, Ranjan A, Covington MF, Ichihashi Y, Fulop D, Jiménez-Gómez JM, Peng J, Maloof JN, et al (2013) A quantitative genetic basis for leaf morphology in a set of precisely defined tomato introgression lines. Plant Cell 25: 2465–2481 23872539 10.1105/tpc.113.112391 PMC3753377

[kiac200-B22] Chung MY , Vrebalov J, Alba R, Lee J, McQuinn R, Chung JD, Klein P, Giovannoni J (2010) A tomato (*Solanum lycopersicum*) *APETALA2/ERF* gene, *SlAP2a*, is a negative regulator of fruit ripening. Plant J 64: 936–947 21143675 10.1111/j.1365-313X.2010.04384.x

[kiac200-B23] Contreras-Lopez O , Moyano TC, Soto DC, Gutierrez RA (2018) Step-by-step construction of gene co-expression networks from high-throughput Arabidopsis RNA sequencing data. Methods Mol Biol 1761: 275–301 29525965 10.1007/978-1-4939-7747-5_21

[kiac200-B24] D’Amelia V , Raiola A, Carputo D, Filippone E, Barone A, Rigano MM (2019) A basic helix-loop-helix (SlARANCIO), identified from a *Solanum pennellii* introgression line, affects carotenoid accumulation in tomato fruits. Sci Rep 9: 3699 30842571 10.1038/s41598-019-40142-3 PMC6403429

[kiac200-B25] Eshed Y , Zamir D (1995) An introgression line population of *Lycopersicon pennellii* in the cultivated tomato enables the identification and fine mapping of yield-associated QTL. Genetics 141: 1147–1162 8582620 10.1093/genetics/141.3.1147 PMC1206837

[kiac200-B26] Fernandez-Moreno JP , Tzfadia O, Forment J, Presa S, Rogachev I, Meir S, Orzaez D, Aharoni A, Granell A (2016) Characterization of a new pink-fruited tomato mutant results in the identification of a null allele of the SlMYB12 transcription factor. Plant Physiol 171: 1821–1836 27208285 10.1104/pp.16.00282 PMC4936558

[kiac200-B27] Fridman E , Carrari F, Liu YS, Fernie AR, Zamir D (2004) Zooming in on a quantitative trait for tomato yield using interspecific introgressions. Science 305: 1786–1789 15375271 10.1126/science.1101666

[kiac200-B28] Fujisawa M , Nakano T, Shima Y, Ito Y (2013) A large-scale identification of direct targets of the tomato MADS box transcription factor RIPENING INHIBITOR reveals the regulation of fruit ripening. Plant Cell 25: 371–386 23386264 10.1105/tpc.112.108118 PMC3608766

[kiac200-B29] Fujisawa M , Shima Y, Higuchi N, Nakano T, Koyama Y, Kasumi T, Ito Y (2012) Direct targets of the tomato-ripening regulator RIN identified by transcriptome and chromatin immunoprecipitation analyses. Planta 235: 1107–1122 22160566 10.1007/s00425-011-1561-2

[kiac200-B30] Gao L , Gonda I, Sun H, Ma Q, Bao K, Tieman DM, Burzynski-Chang EA, Fish TL, Stromberg KA, Sacks GL, et al (2019) The tomato pan-genome uncovers new genes and a rare allele regulating fruit flavor. Nat Genet 51: 1044–1051 31086351 10.1038/s41588-019-0410-2

[kiac200-B31] Gao Y , Wei W, Fan Z, Zhao X, Zhang Y, Jing Y, Zhu B, Zhu H, Shan W, Chen JJ (2020) Re-evaluation of the nor mutation and the role of the NAC-NOR transcription factor in tomato fruit ripening. J Exp Bot 71: 3560–3574 32338291 10.1093/jxb/eraa131 PMC7307841

[kiac200-B32] Giovannoni J , Nguyen C, Ampofo B, Zhong S, Fei Z (2017) The epigenome and transcriptional dynamics of fruit ripening. Annu Rev Plant Biol 68: 61–84 28226232 10.1146/annurev-arplant-042916-040906

[kiac200-B33] Hileman LC , Sundstrom JF, Litt A, Chen M, Shumba T, Irish VF (2006) Molecular and phylogenetic analyses of the MADS-box gene family in tomato. Mol Biol Evol 23: 2245–2258 16926244 10.1093/molbev/msl095

[kiac200-B34] Irfan M , Ghosh S, Meli VS, Kumar A, Kumar V, Chakraborty N, Chakraborty S, Datta A (2016) Fruit ripening regulation of alpha-mannosidase expression by the MADS box transcription factor RIPENING INHIBITOR and ethylene. Front Plant Sci 7: 10 26834776 10.3389/fpls.2016.00010 PMC4720780

[kiac200-B35] Ito Y , Nishizawa-Yokoi A, Endo M, Mikami M, Shima Y, Nakamura N, Kotake-Nara E, Kawasaki S, Toki S (2017) Re-evaluation of the *rin* mutation and the role of RIN in the induction of tomato ripening. Nat Plants 3: 866–874 29085071 10.1038/s41477-017-0041-5

[kiac200-B36] Karlova R , Rosin FM, Busscher-Lange J, Parapunova V, Do PT, Fernie AR, Fraser PD, Baxter C, Angenent GC, de Maagd RA (2011) Transcriptome and metabolite profiling show that APETALA2a is a major regulator of tomato fruit ripening. Plant Cell 23: 923–941 21398570 10.1105/tpc.110.081273 PMC3082273

[kiac200-B37] Kimbara J , Ohyama A, Chikano H, Ito H, Hosoi K, Negoro S, Miyatake K, Yamaguchi H, Nunome T, Fukuoka H, et al (2018) QTL mapping of fruit nutritional and flavor components in tomato (*Solanum lycopersicum*) using genome-wide SSR markers and recombinant inbred lines (RILs) from an intra-specific cross. Euphytica 214: 210

[kiac200-B38] Kumar R , Agarwal P, Tyagi AK, Sharma AK (2012) Genome-wide investigation and expression analysis suggest diverse roles of auxin-responsive *GH3* genes during development and response to different stimuli in tomato (*Solanum lycopersicum*). Mol Genet Genomics 287: 221–235 22228229 10.1007/s00438-011-0672-6

[kiac200-B39] Lanahan MB , Yen HC, Giovannoni JJ, Klee HJ (1994) The never ripe mutation blocks ethylene perception in tomato. Plant Cell 6: 521–530 8205003 10.1105/tpc.6.4.521 PMC160455

[kiac200-B40] Li S , Chen K, Grierson D (2021) Molecular and hormonal mechanisms regulating fleshy fruit ripening. Cells 10: 1136 34066675 10.3390/cells10051136 PMC8151651

[kiac200-B41] Li Y , Chen Y, Zhou L, You S, Deng H, Chen Y, Alseekh S, Yuan Y, Fu R, Zhang Z, et al (2020) MicroTom metabolic network: rewiring tomato metabolic regulatory network throughout the growth cycle. Mol Plant 13: 1203–1218 32561360 10.1016/j.molp.2020.06.005

[kiac200-B42] Liu M , Gomes BL, Mila I, Purgatto E, Peres LE, Frasse P, Maza E, Zouine M, Roustan JP, Bouzayen M, et al (2016) Comprehensive profiling of ethylene response factor expression identifies ripening-associated ERF genes and their link to key regulators of fruit ripening in tomato. Plant Physiol 170: 1732–1744 26739234 10.1104/pp.15.01859 PMC4775140

[kiac200-B43] Lu P , Yu S, Zhu N, Chen YR, Zhou B, Pan Y, Tzeng D, Fabi JP, Argyris J, Garcia-Mas J, et al (2018) Genome encode analyses reveal the basis of convergent evolution of fleshy fruit ripening. Nat Plants 4: 784–791 30250279 10.1038/s41477-018-0249-z

[kiac200-B44] Manning K , Tor M, Poole M, Hong Y, Thompson AJ, King GJ, Giovannoni JJ, Seymour GB (2006) A naturally occurring epigenetic mutation in a gene encoding an SBP-box transcription factor inhibits tomato fruit ripening. Nat Genet 38: 948–952 16832354 10.1038/ng1841

[kiac200-B45] Martel C , Vrebalov J, Tafelmeyer P, Giovannoni JJ (2011) The tomato MADS-box transcription factor RIPENING INHIBITOR interacts with promoters involved in numerous ripening processes in a COLORLESS NONRIPENING-dependent manner. Plant Physiol 157: 1568–1579 21941001 10.1104/pp.111.181107 PMC3252172

[kiac200-B46] McCormick S , Niedermeyer J, Fry J, Barnason A, Horsch R, Fraley R (1986) Leaf disc transformation of cultivated tomato (*L. esculentum*) using *Agrobacterium tumefaciens*. Plant Cell Rep 5: 81–84 24248039 10.1007/BF00269239

[kiac200-B47] Mishra AK , Puranik S, Bahadur RP, Prasad M (2012) The DNA-binding activity of an AP2 protein is involved in transcriptional regulation of a stress-responsive gene, *SiWD40*, in foxtail millet. Genomics 100: 252–263 22771384 10.1016/j.ygeno.2012.06.012

[kiac200-B48] Mutwil M , Klie S, Tohge T, Giorgi FM, Wilkins O, Campbell MM, Fernie AR, Usadel B, Nikoloski Z, Persson S (2011) PlaNet: combined sequence and expression comparisons across plant networks derived from seven species. Plant Cell 23: 895–910 21441431 10.1105/tpc.111.083667 PMC3082271

[kiac200-B49] Ofner I , Lashbrooke J, Pleban T, Aharoni A, Zamir D (2016) *Solanum pennellii* backcross inbred lines (BILs) link small genomic bins with tomato traits. Plant J 87: 151–160 27121752 10.1111/tpj.13194

[kiac200-B50] Orzaez D , Medina A, Torre S, Fernandez-Moreno JP, Rambla JL, Fernandez-Del-Carmen A, Butelli E, Martin C, Granell A (2009) A visual reporter system for virus-induced gene silencing in tomato fruit based on anthocyanin accumulation. Plant Physiol 150: 1122–1134 19429602 10.1104/pp.109.139006 PMC2705029

[kiac200-B51] Orzaez D , Mirabel S, Wieland WH, Granell A (2006) Agroinjection of tomato fruits. A tool for rapid functional analysis of transgenes directly in fruit. Plant Physiol 140: 3–11 16403736 10.1104/pp.105.068221 PMC1326026

[kiac200-B52] Osorio S , Alba R, Damasceno CMB, Lopez-Casado G, Lohse M, Zanor MI, Tohge T, Usadel B, Rose JKC, Fei Z, et al (2011) Systems biology of tomato fruit development: combined transcript, protein, and metabolite analysis of tomato transcription factor (*nor*, *rin*) and ethylene receptor (*Nr*) mutants reveals novel regulatory interactions. Plant Physiol 157: 405–425 21795583 10.1104/pp.111.175463 PMC3165888

[kiac200-B53] Proost S , Krawczyk A, Mutwil M (2017) LSTrAP: efficiently combining RNA sequencing data into co-expression networks. BMC Bioinformatics 18: 444 29017446 10.1186/s12859-017-1861-z PMC5634843

[kiac200-B54] Ramsay NA , Glover BJ (2005) MYB–bHLH–WD40 protein complex and the evolution of cellular diversity. Trends Plant Sci 10: 63–70 15708343 10.1016/j.tplants.2004.12.011

[kiac200-B55] Ranjan A , Budke JM, Rowland SD, Chitwood DH, Kumar R, Carriedo L, Ichihashi Y, Zumstein K, Maloof JN, Sinha NR (2016) eQTL regulating transcript levels associated with diverse biological processes in tomato. Plant Physiol 172: 328–340 27418589 10.1104/pp.16.00289 PMC5074602

[kiac200-B56] Robinson R (1968) Ripening inhibitor: a gene with multiple effects on ripening. Rep Tomato Genet Coop 18: 36–37

[kiac200-B57] Rocha-Sosa M , Sonnewald U, Frommer W, Stratmann M, Schell J, Willmitzer L (1989) Both developmental and metabolic signals activate the promoter of a class I patatin gene. EMBO J 8: 23–29 16453867 10.1002/j.1460-2075.1989.tb03344.x PMC400768

[kiac200-B58] Rockman MV , Kruglyak L (2009) Recombinational landscape and population genomics of *Caenorhabditis elegan*s. PLoS Genet 5: e1000419 19283065 10.1371/journal.pgen.1000419 PMC2652117

[kiac200-B59] Rodriguez-Lopez CE , Hernandez-Brenes C, Trevino V, Diaz de la Garza RI (2017) Avocado fruit maturation and ripening: dynamics of aliphatic acetogenins and lipidomic profiles from mesocarp, idioblasts and seed. BMC Plant Biol 17: 159 28969589 10.1186/s12870-017-1103-6 PMC5623960

[kiac200-B60] Rohrmann J , McQuinn R, Giovannoni JJ, Fernie AR, Tohge T (2012) Tissue specificity and differential expression of transcription factors in tomato provide hints of unique regulatory networks during fruit ripening. Plant Signal Behav 7: 1639–1647 23073014 10.4161/psb.22264 PMC3578905

[kiac200-B61] Rohrmann J , Tohge T, Alba R, Osorio S, Caldana C, McQuinn R, Arvidsson S, van der Merwe MJ, Riano-Pachon DM, Mueller-Roeber B, et al (2011) Combined transcription factor profiling, microarray analysis and metabolite profiling reveals the transcriptional control of metabolic shifts occurring during tomato fruit development. Plant J 68: 999–1013 21851430 10.1111/j.1365-313X.2011.04750.x

[kiac200-B62] Salem MA , Juppner J, Bajdzienko K, Giavalisco P (2016) Protocol: a fast, comprehensive and reproducible one-step extraction method for the rapid preparation of polar and semi-polar metabolites, lipids, proteins, starch and cell wall polymers from a single sample. Plant Methods 12: 45 27833650 10.1186/s13007-016-0146-2 PMC5103428

[kiac200-B63] Sauvage C , Segura V, Bauchet G, Stevens R, Do PT, Nikoloski Z, Fernie AR, Causse M (2014) Genome-wide association in tomato reveals 44 candidate loci for fruit metabolic traits. Plant Physiol 165: 1120–1132 24894148 10.1104/pp.114.241521 PMC4081326

[kiac200-B64] Schauer N , Semel Y, Balbo I, Steinfath M, Repsilber D, Selbig J, Pleban T, Zamir D, Fernie AR (2008) Mode of inheritance of primary metabolic traits in tomato. Plant Cell 20: 509–523 18364465 10.1105/tpc.107.056523 PMC2329927

[kiac200-B65] Semel Y , Nissenbaum J, Menda N, Zinder M, Krieger U, Issman N, Pleban T, Lippman Z, Gur A, Zamir D (2006) Overdominant quantitative trait loci for yield and fitness in tomato. Proc Natl Acad Sci USA 103: 12981–12986 16938842 10.1073/pnas.0604635103 PMC1552043

[kiac200-B66] Shannon P , Markiel A, Ozier O, Baliga NS, Wang JT, Ramage D, Amin N, Schwikowski B, Ideker T (2003) Cytoscape: a software environment for integrated models of biomolecular interaction networks. Genome Res 13: 2498–2504 14597658 10.1101/gr.1239303 PMC403769

[kiac200-B67] Shi Y , Vrebalov J, Zheng H, Xu Y, Yin X, Liu W, Liu Z, Sorensen I, Su G, Ma Q, et al (2021) A tomato LATERAL ORGAN BOUNDARIES transcription factor, *SlLOB1*, predominantly regulates cell wall and softening components of ripening. Proc Natl Acad Sci USA 118: e2102486118 10.1073/pnas.2102486118 PMC8379924 34380735

[kiac200-B68] Shinozaki Y , Nicolas P, Fernandez-Pozo N, Ma Q, Evanich DJ, Shi Y, Xu Y, Zheng Y, Snyder SI, Martin LBB, et al (2018) High-resolution spatiotemporal transcriptome mapping of tomato fruit development and ripening. Nat Commun 9: 364 29371663 10.1038/s41467-017-02782-9 PMC5785480

[kiac200-B69] Sønderby IE , Hansen BG, Bjarnholt N, Ticconi C, Halkier BA, Kliebenstein DJ (2007) A systems biology approach identifies a R2R3 MYB gene subfamily with distinct and overlapping functions in regulation of aliphatic glucosinolates. PLoS ONE 2: e1322 18094747 10.1371/journal.pone.0001322 PMC2147653

[kiac200-B70] Sravankumar T , Naik N, Kumar R (2018) A ripening-induced *SlGH3-2* gene regulates fruit ripening via adjusting auxin-ethylene levels in tomato (*Solanum lycopersicum L.*). Plant Mol Biol 98: 455–469 30367324 10.1007/s11103-018-0790-1

[kiac200-B71] Steinhauser M-C , Steinhauser D, Koehl K, Carrari F, Gibon Y, Fernie AR, Stitt M (2010) Enzyme activity profiles during fruit development in tomato cultivars and *Solanum pennellii*. Plant Physiol 153: 80–98 20335402 10.1104/pp.110.154336 PMC2862428

[kiac200-B72] Szymanski J , Bocobza S, Panda S, Sonawane P, Cardenas PD, Lashbrooke J, Kamble A, Shahaf N, Meir S, Bovy A, et al (2020) Analysis of wild tomato introgression lines elucidates the genetic basis of transcriptome and metabolome variation underlying fruit traits and pathogen response. Nat Genet 52: 1111–1121 32989321 10.1038/s41588-020-0690-6

[kiac200-B73] Tieman D , Zhu G, Resende MF Jr, Lin T, Nguyen C, Bies D, Rambla JL, Beltran KS, Taylor M, Zhang B, et al (2017) A chemical genetic roadmap to improved tomato flavor. Science 355: 391–394 28126817 10.1126/science.aal1556

[kiac200-B74] Tieman DM , Zeigler M, Schmelz EA, Taylor MG, Bliss P, Kirst M, Klee HJ (2006) Identification of loci affecting flavour volatile emissions in tomato fruits. J Exp Bot 57: 887–896 16473892 10.1093/jxb/erj074

[kiac200-B75] Tigchelaar E (1973) A new ripening mutant, non-ripening (nor). Rep Tomato Genet Coop 35: 20

[kiac200-B76] Tomato Genome Consortium (2012) The tomato genome sequence provides insights into fleshy fruit evolution. Nature 485: 635–641 10.1038/nature11119 PMC3378239 22660326

[kiac200-B77] Vallarino JG , Kubiszewski-Jakubiak S, Ruf S, Rossner M, Timm S, Bauwe H, Carrari F, Rentsch D, Bock R, Sweetlove LJ, et al (2020) Multi-gene metabolic engineering of tomato plants results in increased fruit yield up to 23%. Sci Rep 10: 17219 33057137 10.1038/s41598-020-73709-6 PMC7560729

[kiac200-B78] Vrebalov J , Ruezinsky D, Padmanabhan V, White R, Medrano D, Drake R, Schuch W, Giovannoni J (2002) A MADS-box gene necessary for fruit ripening at the tomato *ripening-inhibitor* (*rin*) locus. Science 296: 343–346 11951045 10.1126/science.1068181

[kiac200-B79] Wang L , Zhang XL, Wang L, Tian Y, Jia N, Chen S, Shi NB, Huang X, Zhou C, Yu Y, et al (2017) Regulation of ethylene-responsive *SlWRKYs* involved in color change during tomato fruit ripening. Sci Rep 7: 16674 29192231 10.1038/s41598-017-16851-y PMC5709409

[kiac200-B80] Wang R , Tavano E, Lammers M, Martinelli AP, Angenent GC, de Maagd RA (2019) Re-evaluation of transcription factor function in tomato fruit development and ripening with CRISPR/Cas9-mutagenesis. Sci Rep 9: 1696 30737425 10.1038/s41598-018-38170-6 PMC6368595

[kiac200-B81] Wang S , Lu G, Hou Z, Luo Z, Wang T, Li H, Zhang J, Ye Z (2014) Members of the tomato *FRUITFULL* MADS-box family regulate style abscission and fruit ripening. J Exp Bot 65: 3005–3014 24723399 10.1093/jxb/eru137 PMC4071821

[kiac200-B82] Waseem M , Li N, Su D, Chen J, Li Z (2019) Overexpression of a basic helix–loop–helix transcription factor gene, *SlbHLH22*, promotes early flowering and accelerates fruit ripening in tomato (*Solanum lycopersicum L*.). Planta 250: 173–185 30955097 10.1007/s00425-019-03157-8

[kiac200-B83] Xu C , Min J (2011) Structure and function of WD40 domain proteins. Protein Cell 2: 202–214 21468892 10.1007/s13238-011-1018-1 PMC4875305

[kiac200-B84] Yang L , Huang W, Xiong F, Xian Z, Su D, Ren M, Li Z (2017) Silencing of *SlPL*, which encodes a pectate lyase in tomato, confers enhanced fruit firmness, prolonged shelf-life and reduced susceptibility to grey mould. Plant Biotechnol J 15: 1544–1555 28371176 10.1111/pbi.12737 PMC5698048

[kiac200-B85] Ye J , Wang X, Hu T, Zhang F, Wang B, Li C, Yang T, Li H, Lu Y, Giovannoni JJ, et al (2017) An InDel in the promoter of *Al-ACTIVATED MALATE TRANSPORTER9* selected during tomato domestication determines fruit malate contents and aluminum tolerance. Plant Cell 29: 2249–2268 28814642 10.1105/tpc.17.00211 PMC5635988

[kiac200-B86] Zhang J , Hu Z, Yao Q, Guo X, Nguyen V, Li F, Chen G (2018) A tomato MADS-box protein, SlCMB1, regulates ethylene biosynthesis and carotenoid accumulation during fruit ripening. Sci Rep 8: 3413 29467500 10.1038/s41598-018-21672-8 PMC5821886

[kiac200-B87] Zhang Y , Butelli E, Martin C (2014) Engineering anthocyanin biosynthesis in plants. Curr Opin Plant Biol 19: 81–90 24907528 10.1016/j.pbi.2014.05.011

[kiac200-B88] Zhu G , Wang S, Huang Z, Zhang S, Liao Q, Zhang C, Lin T, Qin M, Peng M, Yang C, et al (2018) Rewiring of the fruit metabolome in tomato breeding. Cell 172: 249–261.e12 29328914 10.1016/j.cell.2017.12.019

