# Correction to: A comparative transcriptomics and eQTL approach identifies *SlWD40* as a tomato fruit ripening regulator

**DOI:** 10.1093/plphys/kiad190

**Published:** 2023-03-30

This is a correction to: Feng Zhu, Sagar Sudam Jadhav, Takayuki Tohge, Mohamed A Salem, Je Min Lee, James J Giovannoni, Yunjiang Cheng, Saleh Alseekh, Alisdair R Fernie, A comparative transcriptomics and eQTL approach identifies *SlWD40* as a tomato fruit ripening regulator, *Plant Physiology*, Volume 190, Issue 1, September 2022, Pages 250–266, https://doi.org/10.1093/plphys/kiac200

In the originally published version of this manuscript, an incorrect affiliation was assigned to author Dr Yunjiang Cheng.

In the PDF numbering for assignment of affiliation for Dr Cheng should read: “^2^,” instead of: “^4^,”.

The online first affiliation for Dr Cheng should read:

“National R&D Center for Citrus Preservation, Key Laboratory of Horticultural Plant Biology, Ministry of Education, Huazhong Agricultural University, Wuhan 430070, China” instead of: “Boyce Thompson Institute for Plant Research, Cornell University, Ithaca, New York 14853, USA”.

These emendations have been made in the article.

