# Supplementary material for: A comparative transcriptomics and eQTL approach identifies SlWD40 as a tomato fruit ripening regulator
Source: Plant Physiol. 2022 May 4;190(1):250–66. doi: 10.1093/plphys/kiac200 (PMC9434188; doi:10.1093/plphys/kiac200)
Supplement: kiac200_Supplementary_Data [file kiac200_supplementary_data.zip › Supplemental Figures.pdf]

No.	Gene ID	Function	VIGS phenotype	
1	Solyc05g015370	Putative TF	Green	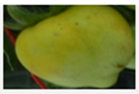
2	Solyc05g015380	Putative TF	Green	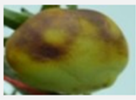
3	Solyc05g015360	Putative TF	Green	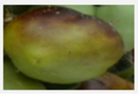
4	Solyc03g044460	BHLH TF	Green	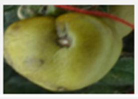
5	Solyc11g010710	AP2-like ethylene-responsive TF	Green	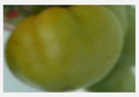
6	Solyc12g009050	CCAAT box binding factor	Green	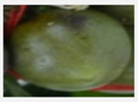
7	Solyc02g090310	C2C2(Zn) DOF zinc finger	Light red	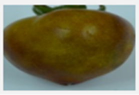
8	Solyc12g098620	BHLH TF	Light red	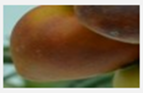
9	Solyc01g080410	Peptide msrB 5	Red	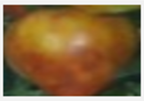
10	Solyc01g094080	Cytochrome P450	Red	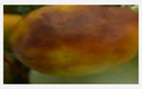
11	Solyc03g116440	Zinc finger, FYVE/PHD-type	Red	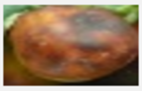
12	Solyc07g066160	C2H2 zinc finger	Red	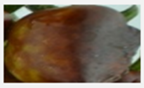
13	Solyc03g095900	2-ODD	Red	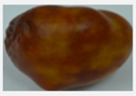
14	Solyc04g005020	WD-40 repeat family protein	Yellow	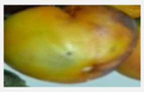
15	Solyc07g052700	MADS-box	Yellow	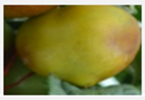
16	Solyc12g010950	Alcohol Dehydrogenase	White Green	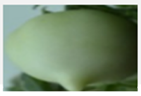

Supplemental Figure S1 VIGS phenotype of candidate genes.

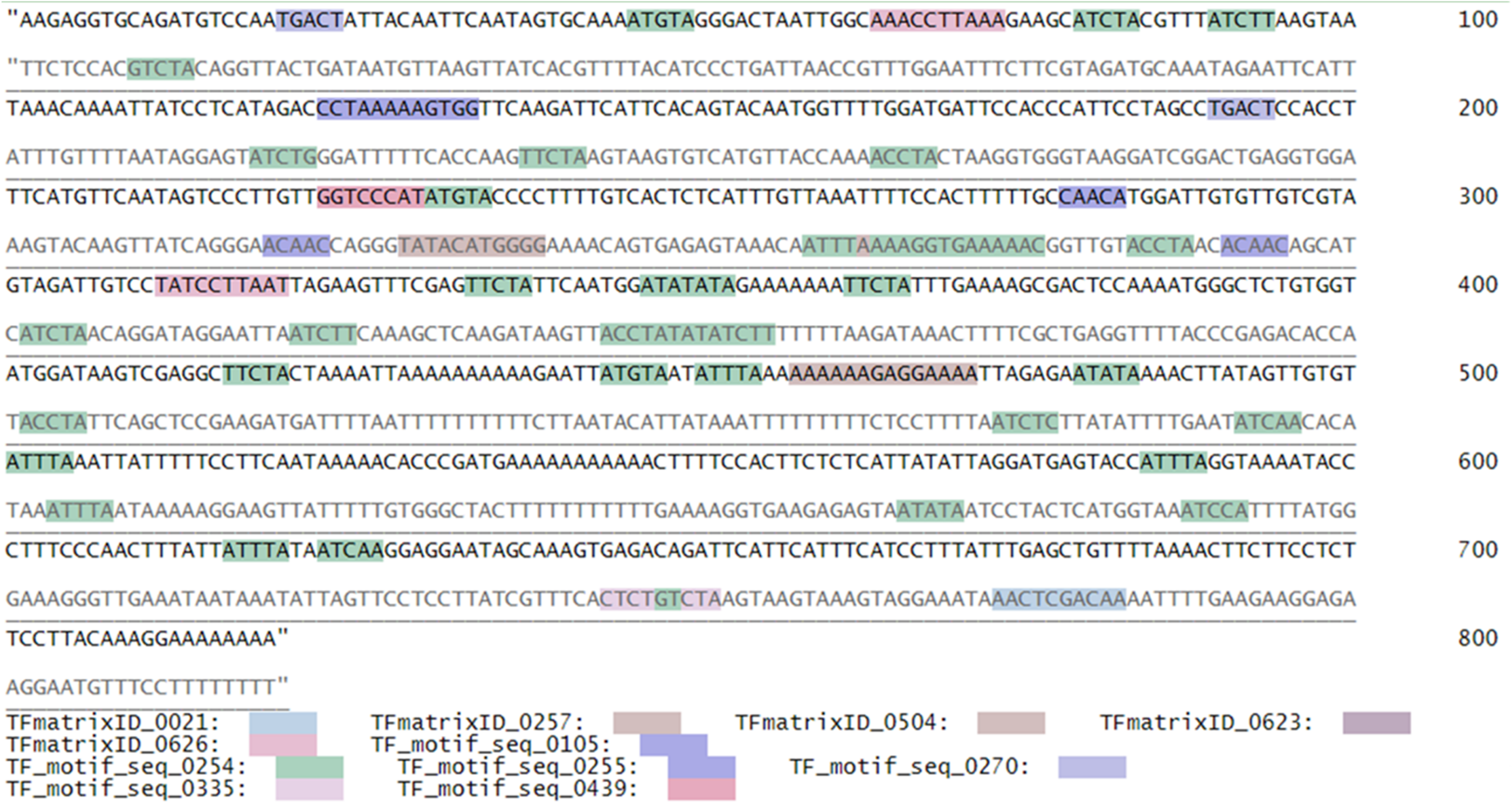

Supplemental Figure S2: Promoter analysis of *SlWD40* for the presence of ethylene (C2H2, AP2 , EIN), auxin and MADS-box binding related cis-regulatory elements. The analysis was performed using 0.7-kb upstream (from start codon of *SlWD40*) sequence. TFmatrixID_0021: C2H2, TFmatrixID_0257: EIN3, TFmatrixID_0504: MADS box; MIKC TFmatrixID_0623: AP2, TFmatrixID_0626: AP2, TFmatrixID_0105: MADS box; MIKC, TFmatrixID_0254: AP2; ERF, TFmatrixID_0255: AP2 ;RAV ;B3, TFmatrixID_0270: WRKY, TFmatrixID_0335: B3 ; ARF, TFmatrixID_0439: Aux/IAA

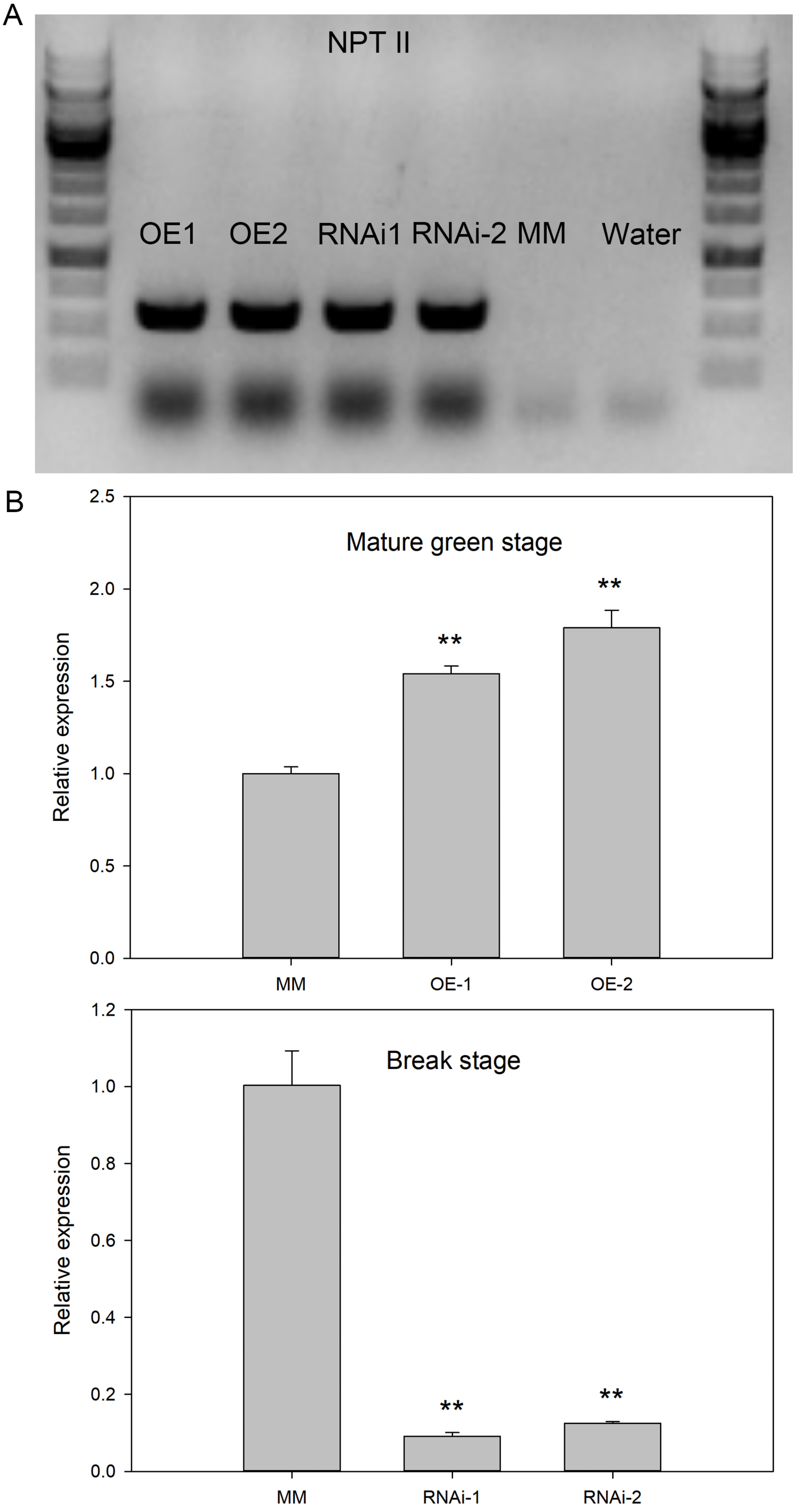

Supplemental Figure S3 The NPT-II specific PCR and real time PCR of *SlWD40* of T0 transformants of RNAi and OE lines. The values in each column are the mean of three biological replicates. Error bars indicate SD. The asterisks indicate statistically significant differences determined by the Student's *t*-test (two-tail): **, $p < 0.01$.
